# Stemodane Diterpenes and Diterpenoids: Isolation, Structure Elucidation, Biogenesis, Biosynthesis, Biological Activity, Biotransformations, Metabolites and Derivatives Biological Activity, Rearrangements †

**DOI:** 10.3390/molecules26092761

**Published:** 2021-05-07

**Authors:** Francesca Leonelli, Luisa Maria Migneco, Alessio Valletta, Rinaldo Marini Bettolo

**Affiliations:** 1 Dipartimento di Chimica, Università degli Studi La Sapienza, Piazzale Aldo Moro 5, I-00185 Roma, Italy; 2 Dipartimento di Biologia Ambientale, Università degli Studi La Sapienza, Piazzale Aldo Moro 5, I-00185 Roma, Italy

**Keywords:** stemodane, diterpenes, diterpenoids, isolation, structure, bioactivity, biotransformations, biogenesis, biosynthesis

## Abstract

The scientific activity carried out over forty-five years on stemodane diterpenes and diterpenoids structure elucidation, biogenesis, biosynthesis, biological activity and biotransformations was reviewed.

## 1. Introduction

**Figure 1:**
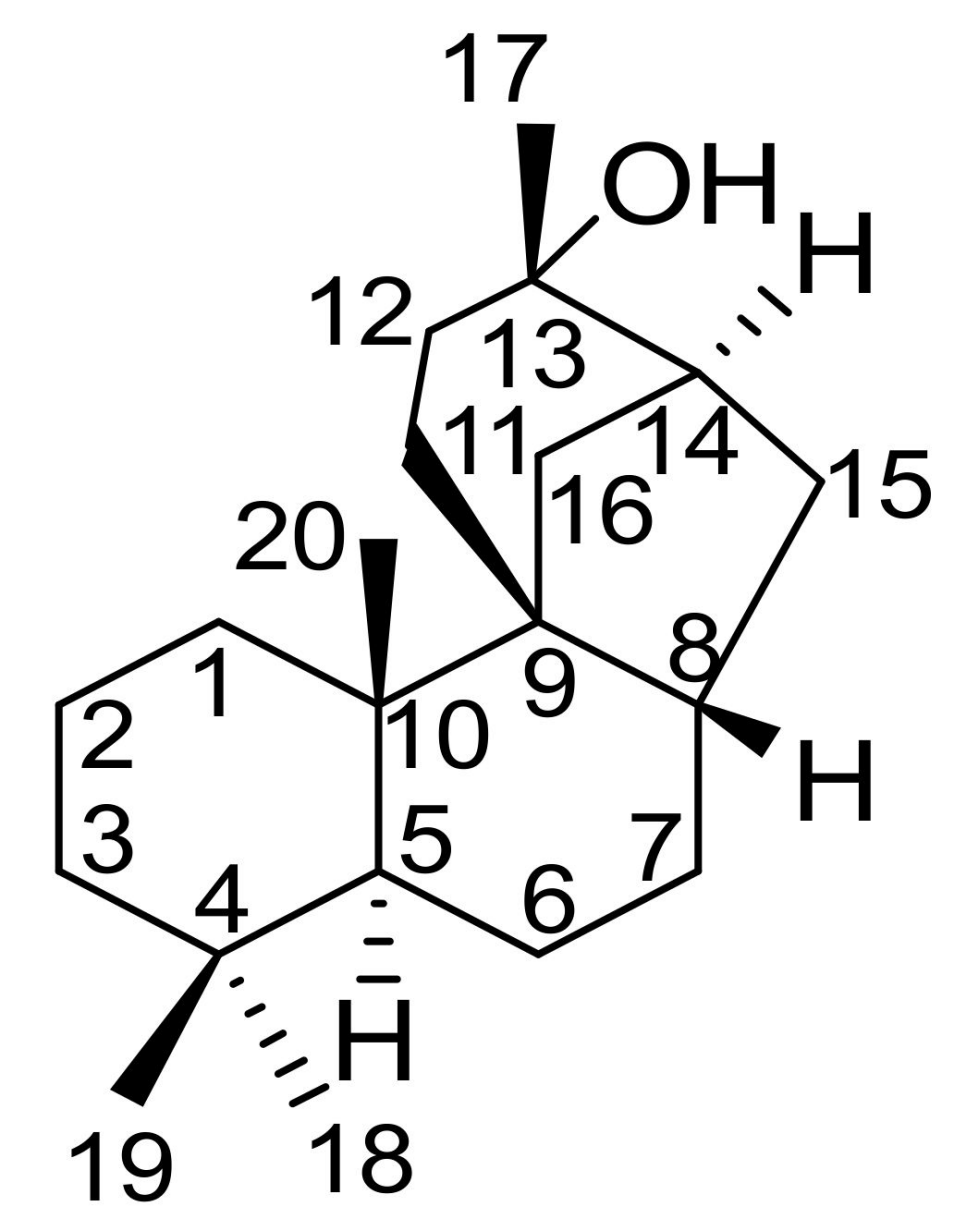
Stemodane diterpenes C-atom skeleton and C(13) substitution.

Stemodane diterpenes and diterpenoids are tetracyclic compounds characterized by the unique hydrocarbon skeleton reported in Figure 1. The main structural features are: C/D ring constituted by a bicycle[3.2.1]octane system fused to the *trans* A/B ring system; two contiguous quaternary carbon atoms, C(9) and C(10), the former spirocyclic; H–C(8) *syn* to the CH_3_–C(10) and to the three-carbon bridge connecting C–(9) and C–(14); HO–C(13) *exo* configurated to the bicyclo[3.2.1]octane system 2-C atom bridge; oxygenated functions may be present at C(2), C(3), C(14), C(17), C(18) and C(19).

## 2. Isolation and Structure Elucidation

**Figure 2:**
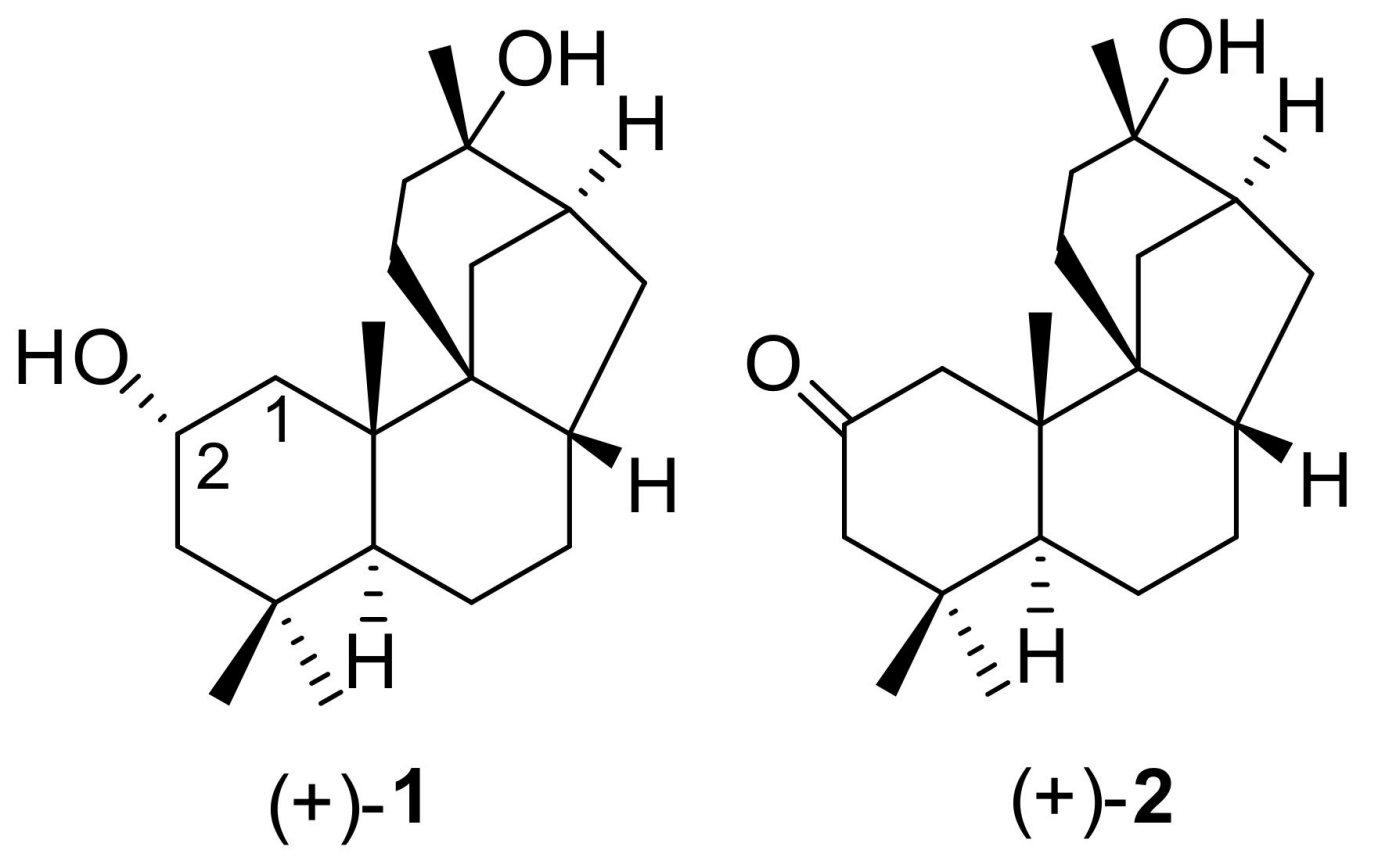
(+)-Stemodin (**1**) and (+)-stemodinone (**2**).

**Figure 3:**
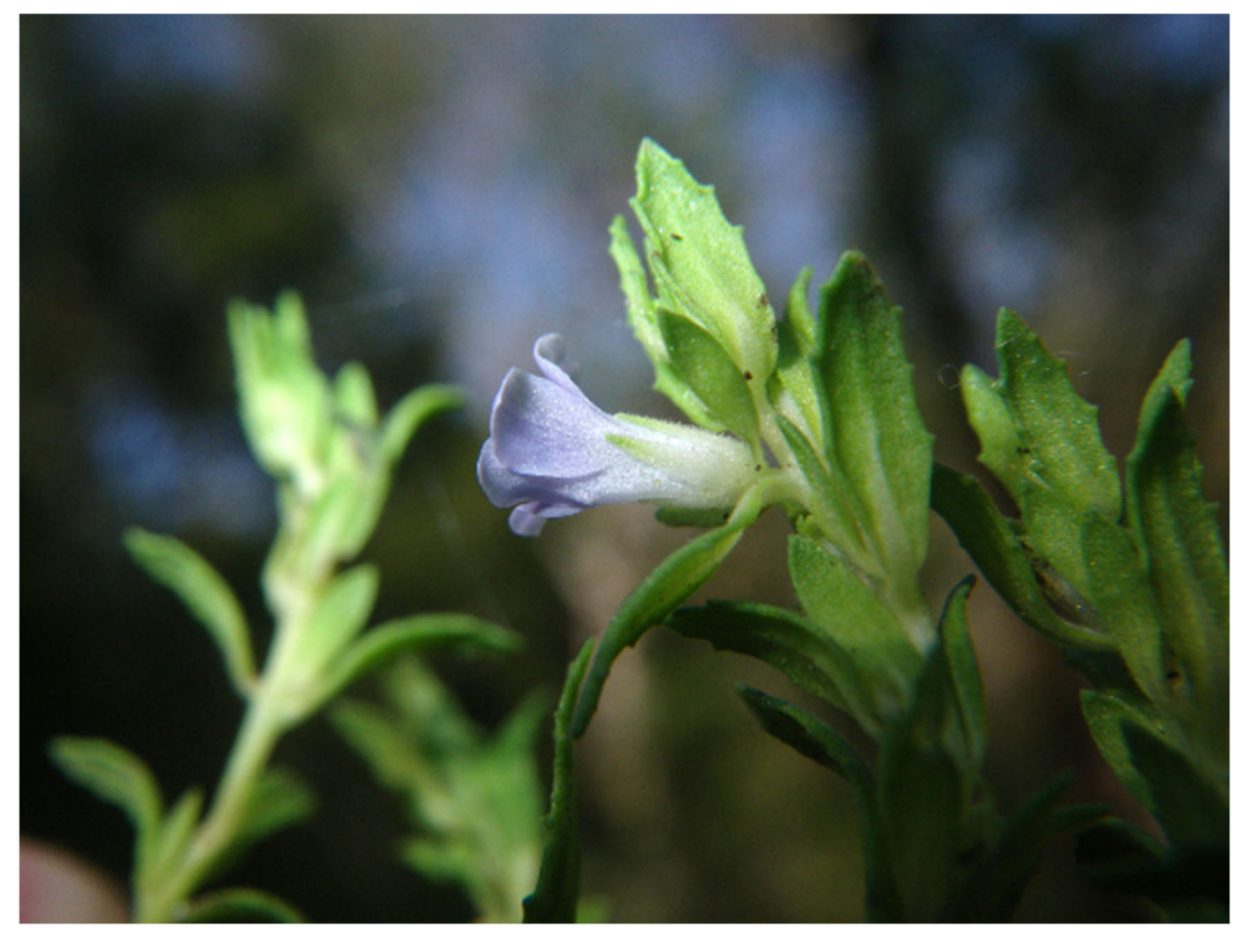
*Stemodia maritima* (Scrophulariaceae).

The isolation of (+)-stemodin (1) and (+)-stemodinone (2) (Figure 2) from the leaves of *Stemodia maritima* L. (Scrophulariaceae) (Figure 3), collected in the Palisadoes peninsula of Jamaica, was described in 1973 by P.S. Manchand (Hoffmann-La Roche Inc., Nutley, N.J.), J.D. White (Oregon State University, Corvallis), H. Wright and J. Clardy (Ames Laboratory-USAEC and Chemistry Dept., Iowa State University) [1].

**Figure 4:**
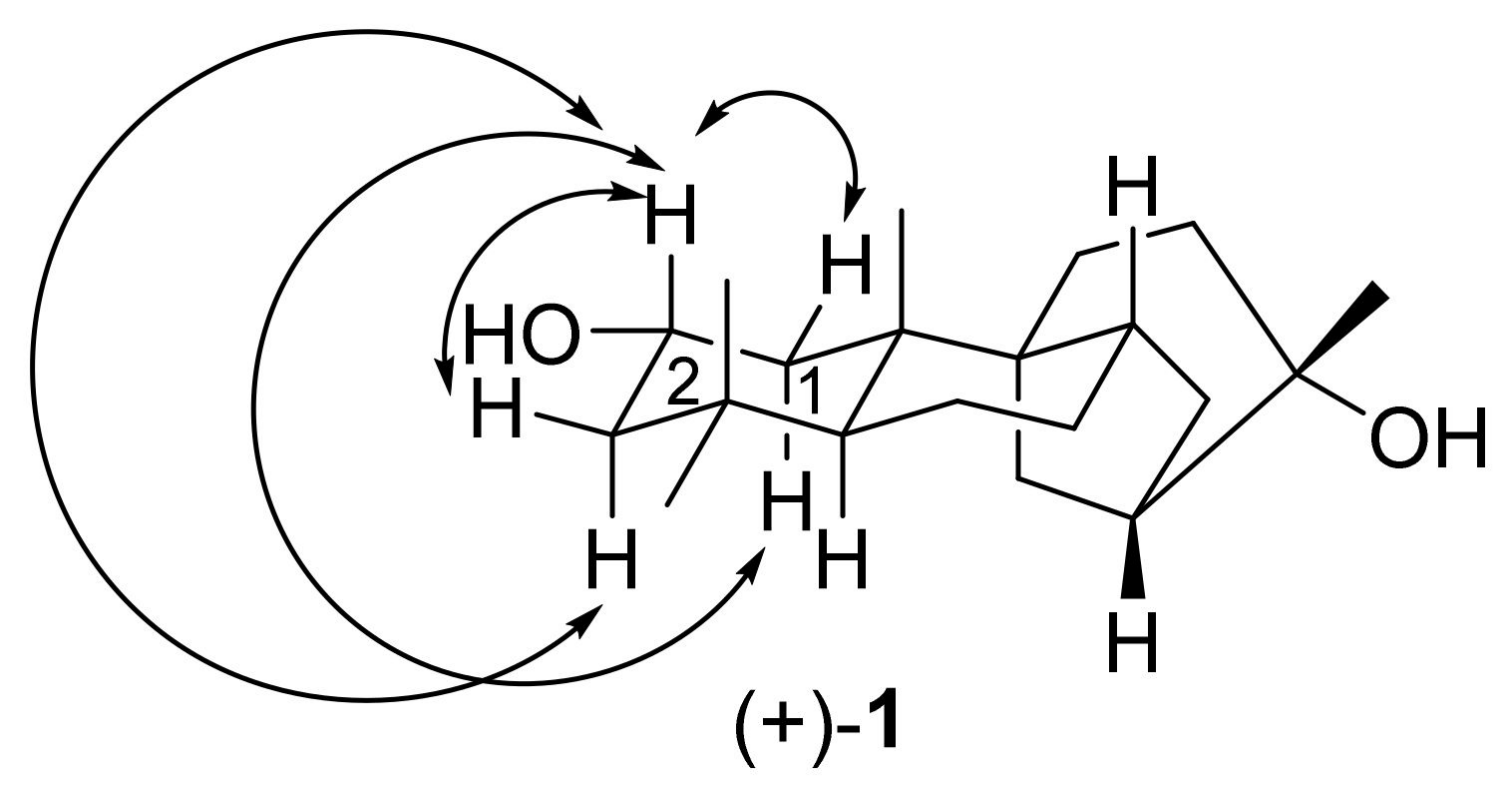
Vicinal coupling of H–C(2) in (+)-stemodin (**1**) [1].

The structures of (+)-**1** and (+)-**2** were established by means of chemical work, IR and ^1^H-NMR, and X-ray diffraction. Thus, Jones oxidation of (+)-**1** to (+)-**2** revealed the relationship between the two compounds. A single-crystal X-ray analysis of (+)-**2** disclosed the novel stemodane tetracyclic ring system. The relative configuration of the HO–C(2) in (+)-**1**, flanked by two methylene groups, was confirmed by ^1^H-NMR spectroscopy. The large coupling constant of the geminal H-C(2) (1H, δ (ppm) = 3.71, t of t, *J* = 11; 3.5 Hz) showed, in fact, that this proton is axial (Figure 4). As a consequence, the geminal HO–C(2) was set equatorial.

The (+)-stemodinone (**2**) absolute configuration was attributed on the basis of its positive ORD Cotton effect. From it followed that of (+)-stemodin (**1**).

A few years later, in 1976, N.J. Doorenbos (University of Mississippi), reported, in collaboration with C.D. Hufford and R.O. Guerrero, the isolation from the aboveground portion of *S. maritima*, collected in the Caribbean island of Curaçao, along with (+)-**1** and (+)-**2**, of (+)-maritimol (**3**) and what was supposed to be (+)-stemodinol (**4**) [2] (vide infra).

The structure of (+)-**3** was deduced by means of chemical work (highlighted below), IR and ^1^H-NMR.

**Scheme 1:**
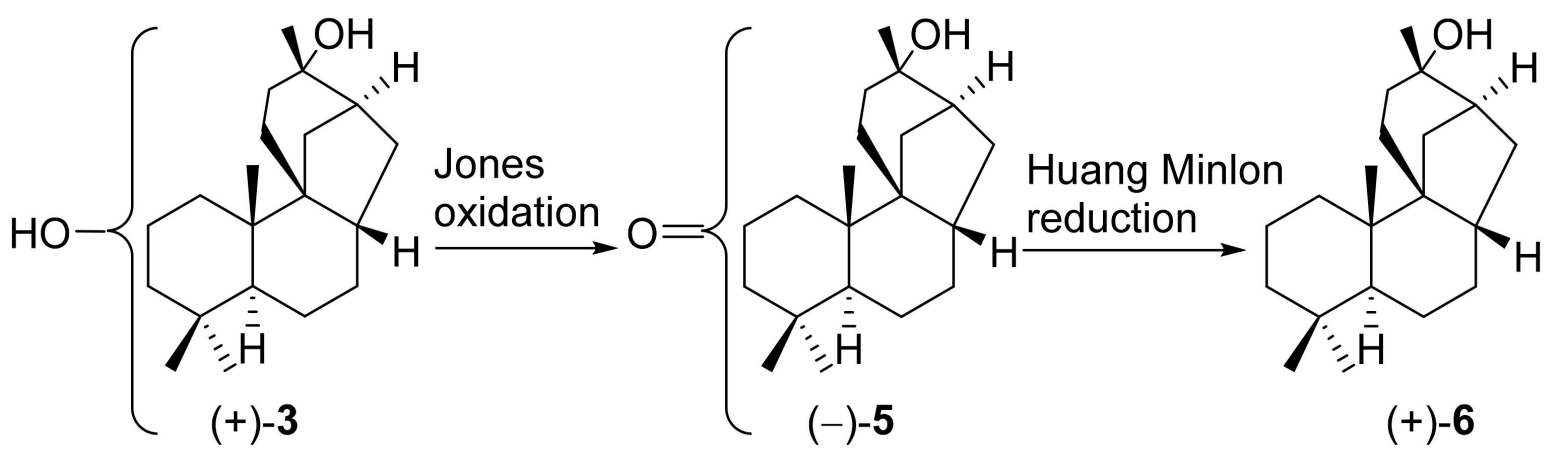
Chemical work for the establishment of (+)-maritimol (**3**) structure [2].

Thus, (+)-**3**, the molecular formula, IR and ^1^H-NMR, of which revealed the absence of unsaturations and the presence of four cycles and two hydroxyls, was oxidized to (−)-**5** and the latter deoxygenated by the Huang–Minlon procedure to give known (+)-2-deoxystemodinone (**6**) [1], thus confirming the stemodane skeleton (Figure 1), the C–(13) substitution and the presence of a secondary hydroxyl (Scheme 1).

**Figure 5:**
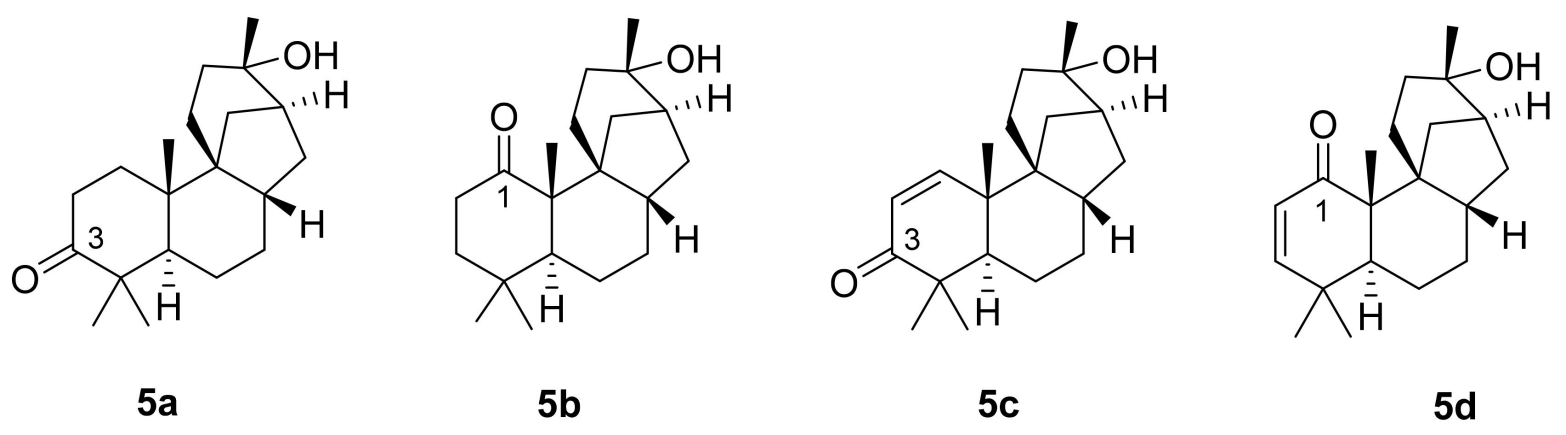
Chemical work for the establishment of (+)-maritimol (**3**) structure [2].

The location of the secondary hydroxy group followed the observation that (−)-**5** could be converted into an α,β-unsaturated ketone. It followed that the carbonyl should have been located at C(1) or at C(3). No other location of the carbonyl group could have accommodated such unsaturated functions (**5c** or **5d**) within the molecule (Figure 5).

**Figure 6:**
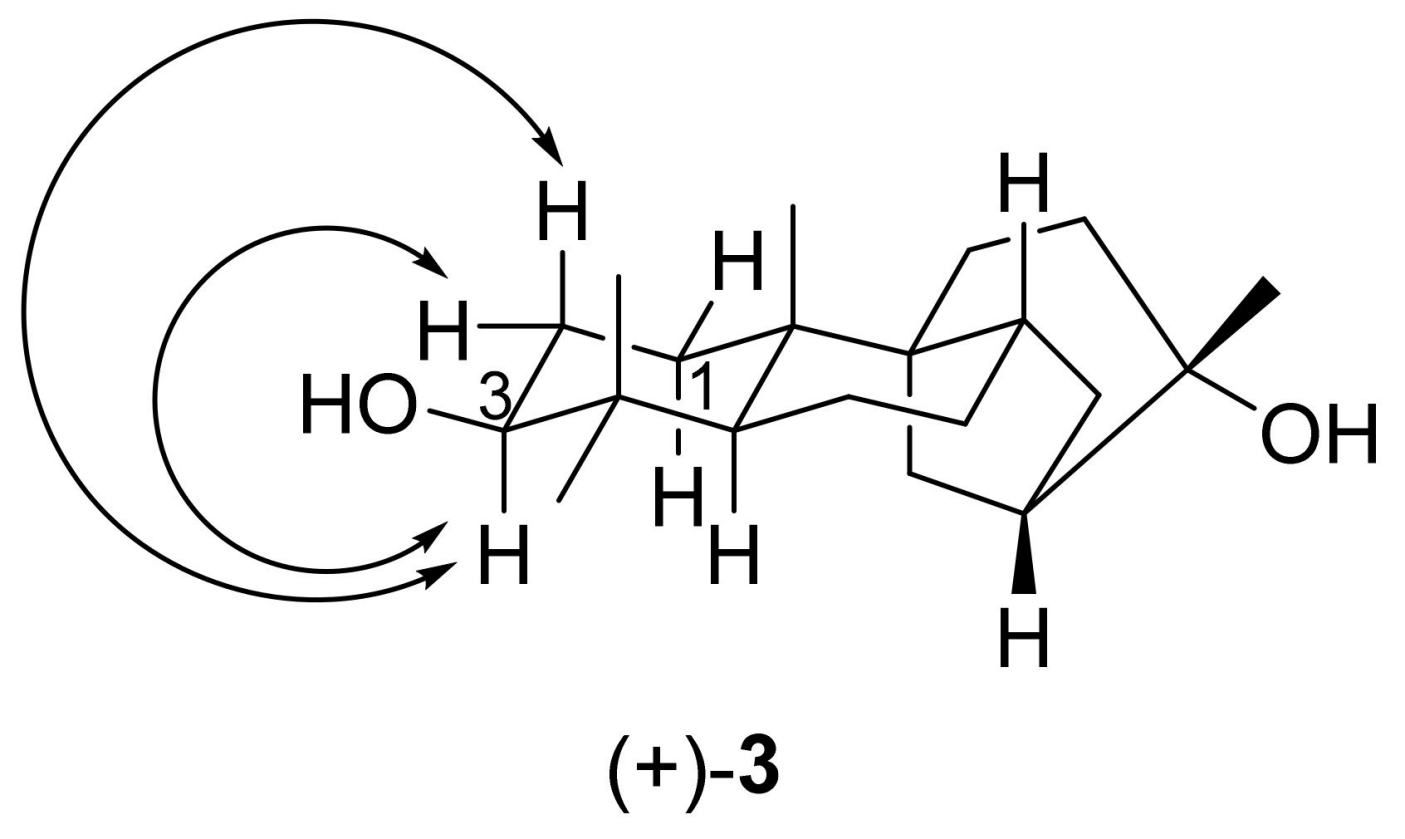
Vicinal coupling of H–C(3) in (+)-maritimol (**3**) [2].

**Figure 7:**
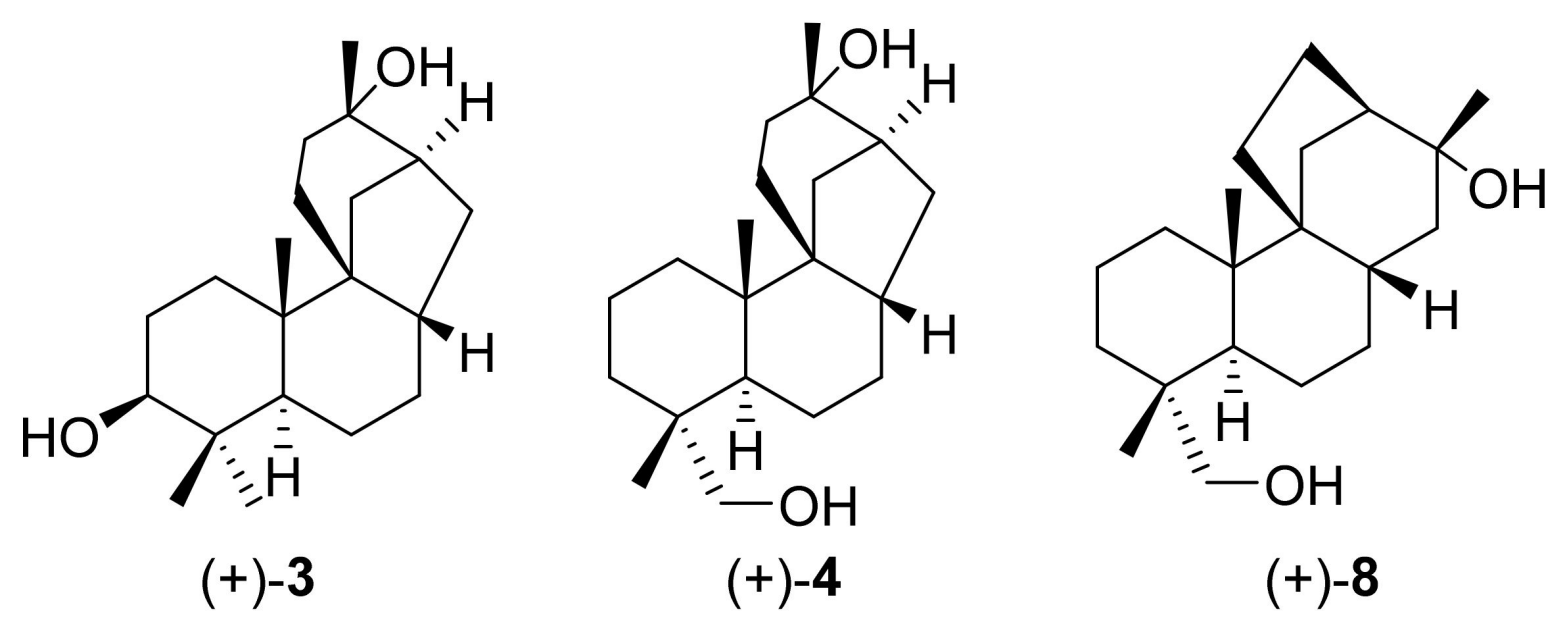
(+)-Maritimol (**3**), (+)-stemodinol (**4**) and (+)-stemarin (**8**).

Applying the octant rule to the corresponding saturated ketones **5a** and **5b,** it was possible to eliminate the location of the carbonyl function at C(1). In fact, for a carbonyl at C(1), the Cotton effect would have been positive, while for a carbonyl at C(3) it would have been negative. The recorded Cotton effect for (−)-**5** was negative. The secondary hydroxy group was therefore placed at C(3). The stereochemistry of the HO–C(3) was established β (equatorial) in view of the large geminal H–C(3) coupling constant (1H, dd, *J* = 11; 5 Hz), indicating that the latter proton was axial (Figure 4). On these grounds, the (+)-maritimol structure (+)-**3** (Figure 6 and Figure 7) was therefore attributed [2].

**Scheme 2:**
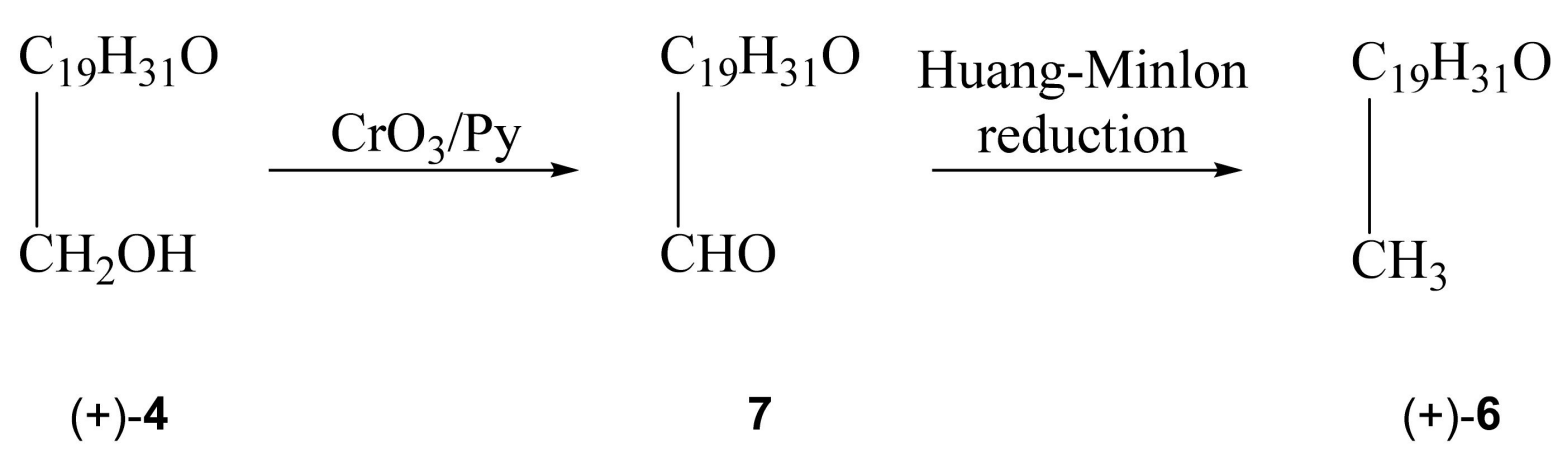
Chemical work for the establishment of (+)-stemodinol (**4**) structure [2].

The same group then addressed the structure elucidation work of what was supposed to be (+)-stemodinol (**4**). The investigation on this new diterpenoid (m.p. 182–183 °C, [α]_d_ = +13.8) was carried out by means of chemical work (highlighted below, Scheme 2), IR and ^1^H-NMR. The ^1^H-NMR spectra revealed the presence of a primary alcohol function that was located at C(18) on the basis of a detailed ^1^H-NMR analysis. In order to confirm the stemodane skeleton, (+)-**4** was oxidized to **7** ([α]_D_ not reported as in all cases where the rotation sign is not given) and the latter deoxygenated by the Huang–Minlon procedure to (+)-**6**. This resulting material was “identical in all respects” with (+)-2-deoxystemodinone (**6**), previously obtained from (+)-**2** and (+)-**3** (*vide supra*), thus confirming the stemodane skeleton and the HO–C(13). Unfortunately, the comparison was misleading. Some years later (*vide infra*), it was, in fact, demonstrated that the structure (+)-**4** had been erroneously attributed to (+)-stemodinol and that the true structure of the latter was that of (+)-stemarin (**8**) (m.p. 183–184 °C, [α]_d_ = +17.8), a structural diastereoisomer with a new and unique skeleton, the isolation from *S. maritima* and structure elucidation of which had been reported by Manchand and Blount in 1975. The paper [2] by Doorenbos and colleagues was submitted to the *J. Pharm. Sci.* at the end of March 1975 and was accepted for publication on Feb. 1976. On Aug. 1975, Manchand and colleagues submitted the paper to *J. C. S. Chem. Commun.* [3], and published in the same year, in which the isolation, X-ray structure and absolute configuration determination of (+)-stemarin (**8**), “a diterpene with a new skeleton” isolated, along with (+)-2-deoxystemodinone (**6**), from *S. maritima* was described. [3] (Figure 7).

Some years later, in 1982, R.B. Kelly (University of New Brunswick, St. John, N.B.) and colleagues, who had previously described the total synthesis of (±)-stemarin (**8**) (*vide infra*), and Manchand, in the frame of their work on stemodane diterpenes, described the preparation of (±)-stemodinol (**4**). The comparison of the synthetic compound with the nominal identical compound isolated by Doorenbos and colleagues [2] showed the two materials were not the same. On the contrary, the comparison with an authentic sample of (+)-stemarin (**8**) showed its identity with the latter [4,5].

We decided to highlight the above work because it nicely represents the structure elucidation of new and complex natural products, such as stemodane diterpenoids, by the tools available in the middle of the seventies.

As will be reported in the sequel, also (+)-stemodinol (**4**) was eventually isolated from *S. maritima*. Its structure was established by HR-ESI-MS and 2D-NMR [6].

**Figure 8:**
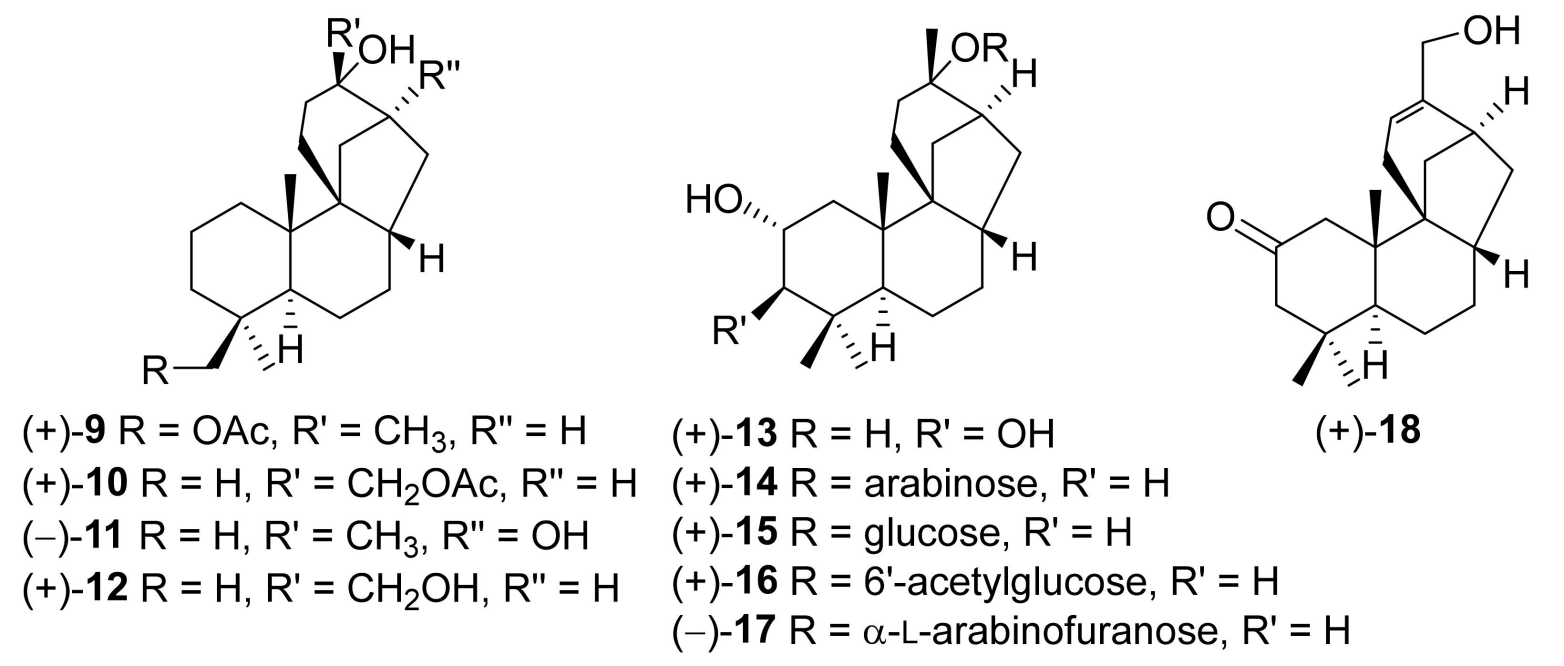
*S. maritima* stemodane diterpenoids **9**–**18**.

In 1988, Hufford reported the complete ^1^H- and ^13^C-NMR assignments for (+)-**1**, (+)-**2** and (+)-**3** [7]. Some years later, in 1991, J.A. Garbarino and colleagues (Universidad Técnica Federico Santa Maria, Valparaiso) reported the isolation from the fresh aerial parts of *Stemodia chilensis* Benth., a plant that grows in humid places in littoral Central Chile, of (+)-2-deoxystemodinone (6) [8]. From the same plant, the Valparaiso group obtained also stemodane diterpenoids **9**–**12** [8] (Figure 8) the structures of which were established by ^1^H-NMR and ^13^C-NMR.

The proposed structure for (+)-**9** [8] was later confirmed by our group with a synthesis (*vide infra*) from (+)-podocarpic acid and a key-intermediate X-ray structure determination [9].

In 1992, Hufford also described the isolation of (+)-maristeminol (**13**), and (+)-stemodinoside A (**14**), (+)-stemodinoside B (**15**) and (+)-stemodinoside C (**16**) from *S. maritima* [10]. The structure of (+)-**13** was established by LC/MS and by comparison of the ^1^H-NMR and ^13^C-NMR spectra with those of (+)-stemodin (**1**) and congeners previously reported [7]. In all compounds, the sugar moiety resulted, attached to the HO–C(13). Acid hydrolysis indicated that the aglycone moiety is in all cases constituted by (+)-stemodin (**1**). The sugar moiety in (+)-stemodinoside A (**14**) is constituted by arabinose, while that of (+)-stemodinoside B (**15**) is constituted by glucose. Finally, the sugar moiety in (+)-stemodinoside C (**16**) is 6′-acetyl glucose. The acetate group was located at C-6′ on the basis of the ^1^H- and ^13^C-NMR H–C(6′) shifts as compared with those of (+)-**15**. Mild basic hydrolysis of (+)-stemodinoside B (**16**) gave (+)-stemodinoside A (**15**). The authors did not establish the absolute configurations of the sugar moieties. Nevertheless, they hypothesized that they could be l-arabinose and d-glucose, the naturally occurring forms in most plants. Furthermore, since in both glycosides the anomeric proton resonates as a doublet (*J* *≅* 7 Hz) the authors hypothesized that (+)-stemodinoside A (**14**) is an α-l-arabinoside, while (+)-stemodinoside B (**15**) and (+)-stemodinoside C (**16**) are β-d-glucosides [10]. The isolation of (+)-maristeminol (**13**) from *Homalomena occulta* (Lour.) Schott was also reported in 2007 by S.-D. Luo and colleagues (State Key Laboratory of Phytochemistry and Plant Resources in West China) [11]. The structure of (+)-**13** was established by comparison of its physicochemical, NMR and MS data with those reported in the literature [10].

Later, in the frame of their work on stemodane diterpenoids, P.B. Reese (University of West Indies, Mona) and colleagues isolated a new compound from *S. maritima* (−)-stemodinoside D (stemodin-α-l-arabinofuranoside) (**17**). Its structure was established by HRMS(EI) and 2D-NMR [12]. Furthermore, in 2010, Arriaga and colleagues (Universidad Federal do Ceará) isolated stemodin 1 from the leaves of *S. maritim*a collected in the Ceará coast in the northeast region of Brazil [13]. Its structure was established by EIMS, IR, ^1^H-NMR and ^13^C-NMR. The specific rotation was not reported. Later, in 2014, the above Brazilian group described the isolation of stemodinol (**4**), stemodin (**1**) and stemodinoside B from *S. maritima* (**15**) [6]. This was the first report about the isolation of (+)-stemodinol (**4**) from natural sources. The structures of **4**, **1** and **15** were elucidated by means of HR-ESI-MS, ^1^H-, ^13^C-NMR and 2D-NMR. In this case, the specific rotations were also not reported. Finally, in 2018 Fu and colleagues (Hainan Normal University and Chinese Academy of Sciences) described the isolation from the stems and leaves of *Trigonostemon heterophyllus* Merr., among other products, of (+)-trigoheterone A (**18**), a new stemodane diterpenoid. The structure of the latter was established on the basis of extensive spectral analyses [14] (Table 1).

## 3. Biogenesis

**Scheme 3:**
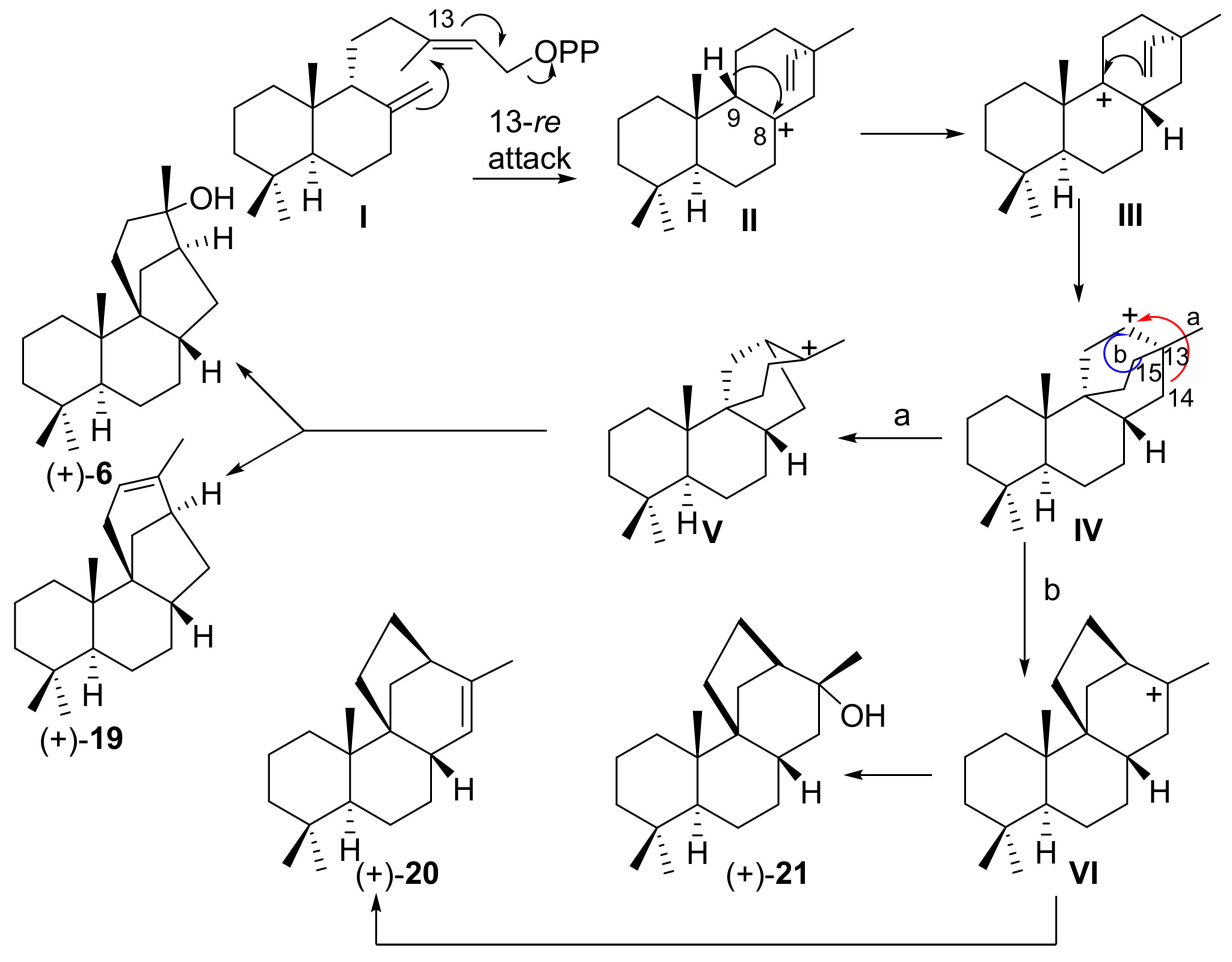
Hypothesized biogenesis of stemodane and stemarane diterpenes and diterpenoids.

The probable biogenesis of stemodane diterpenes and diterpenoids is outlined in Scheme 3. The initial cyclization of *syn*-copalyldiphosphate (*syn*-CPP) (**I**) by *re*-face attack on C(13) leads to the isopimarenyl carbonium ion intermediate **II** [15], which undergoes a C(9) → C (8) 1,2-hydride shift from the β face. The resulting C(9) carbonium ion intermediate **III** cyclizes to the bicyclo[2.2.2]octane carbonium ion intermediate **IV,** which rearranges (migration of the C(13)–C(14) bond through the bottom face, red arrow) to form the stemodanyl carbonium ion intermediate **V**. Deprotonation of the latter affords (+)-stemod-12-ene (**19**) while hydration gives (+)-2-deoxystemodin (**6**) [16,17].

The bicyclo[2.2.2]octane carbonium ion intermediate **III** may also evolve in a different way (blue arrow): migration of the C(13)–C(15) bond through the upper face leads to the stemaranyl carbonium ion intermediate **VI**. Deprotonation of the latter affords (+)-stemar-13-ene (**20**) while hydration gives (+)-18-deoxystemarin (**21**) [16,17].

It is noteworthy that stemodane and stemarane diterpenes and related diterpenoids were both isolated from *S. maritima* [1,3] (*vide supra*). The obtaining of a bicyclo[3.2.1]octane system from a bicyclo[2.2.2]octane intermediate was proposed independently in 1956 by Z. Valenta and K. Wiesner (University of New Brunswick, Fredericton N.B) [18,19] and R.C. Cookson and M.E. Trevett (Birkbeck College, London) [20] for the *Aconitum* L. diterpene alkaloids biogenesis.

**Scheme 4:**
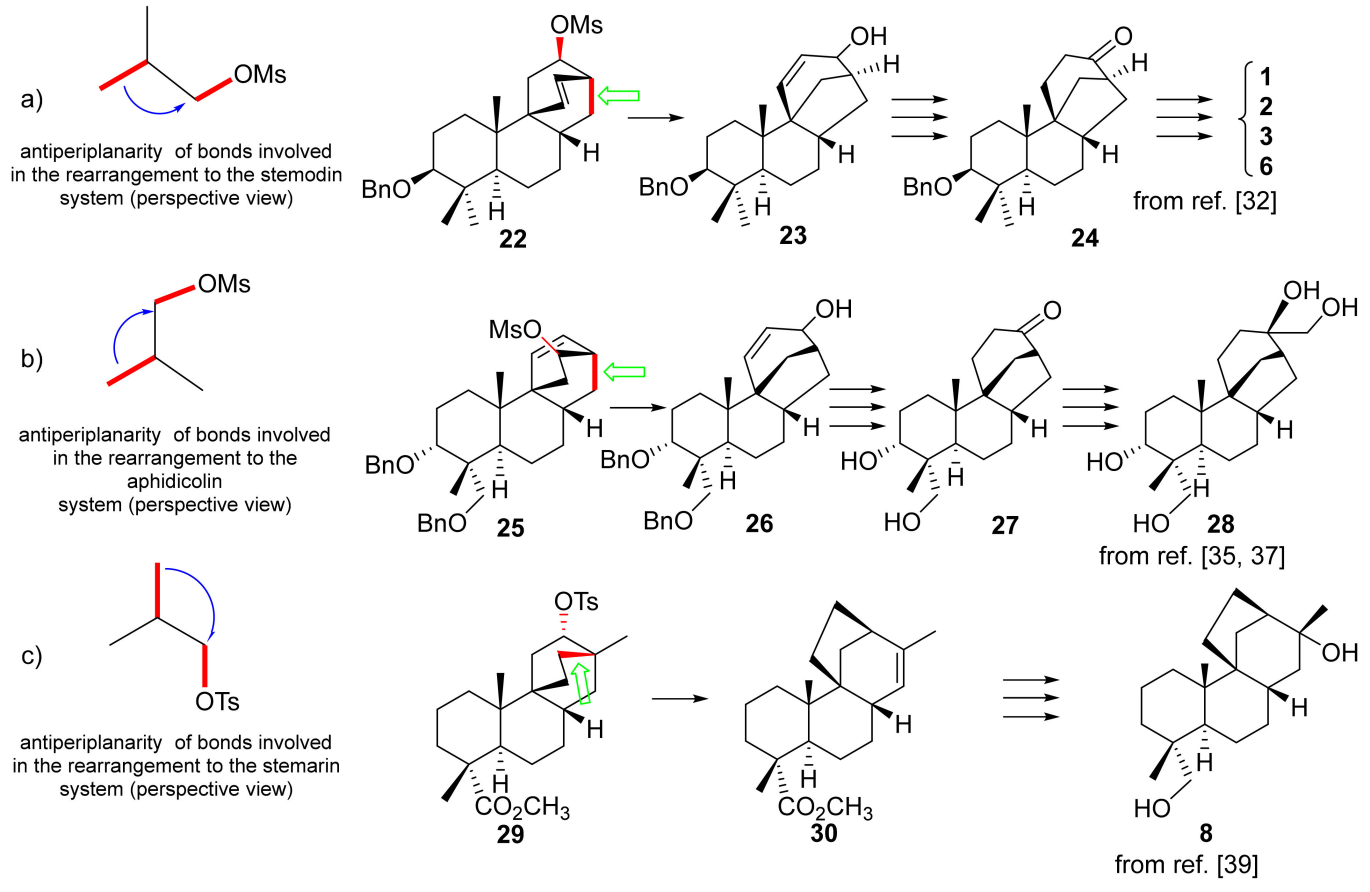
Stereospecificity of bicyclo[2.2.2]octane → bicyclo[3.2.1]octane rearrangements.

Later, Wiesner and his group exploited this rearrangement for the synthesis of several diterpene alkaloids whose C/D ring system is constituted by a bicyclo[3.2.1]octane system [21,22,23,24,25,26,27,28]. This goal was achieved by locating, in a substituted bicyclo[2.2.2]octane system, at a specific position, a suitably configurated leaving group the departure of which promoted the migration of the antiperiplanar C–C bond. Remarkable examples of such specificity were reported in the past in the frame of biogenetic syntheses of stemodane [4,5,9,29,30,31,32,33], aphidicolane [30,31,34,35,36,37,38] and stemarane [39,40,41,42,43,44], diterpenes and diterpenoids. As can be seen (Scheme 4), the bonds indicated by a green arrow migrate in one case (entry a) through the lower face of the molecule, while in entry b, the same bond migrates through the lower face owing to the different location of the leaving group. From entry c in Scheme 4, it can also be appreciated (entry c vs. a) how a different orientation of the leaving group induces the migration of a different C–C bond.

The mechanistic aspects of the bicyclo[2.2.2]octane → bicyclo[3.2.1]octane rearrangement have also been studied on simpler systems by H.M. Walborsky, M.E. Baum and A.A. Youssef (Florida State University, Tallahassee) [45], H.L. Goering and colleagues (University of Wisconsin, Madison) [46,47,48] and Kraus and colleagues (Tübingen University) [49]. They also observed that the nucleophilic group attacks the intermediate carbonium ion in such a way that the incoming nucleophile results afterward as *exo* configurated to the bicyclo[3.2.1]octane two C-bridge.

Having observed that in diterpenoids, whose C/D ring system is constituted by a bicyclo[3.2.1]octane moiety, the HO group at the former bridge-head C-atom is always *exo* configurated with respect to the two C-atom bridge and in view of the above-quoted Walborsky and Goering work [45,46,47,48], a biogenetic synthesis of (±)-2-deoxystemodinone (**6**), in which the desired (natural) substitution at C(13) was obtained in a 7:1 ratio over the C(13) epimer, was achieved in the past by our group [33].

These types of rearrangements have also been recently the object of a quantum chemistry investigation by Y.J. Hong and D.J. Tantillo (University of California-Davis) [50]. Their work clarified the mechanisms of formation from *syn*-CPP of stemodane diterpenes and related diterpenes. The complex network of reaction pathways involving concerted but asynchronous dyotropic and triple shift rearrangements was disclosed.

## 4. Biosynthesis

Differently from structurally related tetracyclic diterpenoid (+)-aphidicolin (28), an antimitotic and antiviral metabolite produced by the fungus *Cephalosporium aphidicola* Petch [51,52], whose biosynthesis was investigated by radioisotope and ^13^C-NMR methods, revealing the origin of the carbon skeleton [53] and by incorporation of labelled intermediates [54], to our knowledge, similar studies were not carried out for stemodane diterpenes and diterpenoids.

Nevertheless, the synthases responsible for their biosynthesis, which occurs in plastids, were investigated. As known, diterpene synthases are encoded by nuclear genes, which are imported into the plastids after being synthesized in the cytoplasm thanks to cleavable N-terminal transit peptides [55,56]. Geranylgeranyl diphosphate (GGPP) (**VII**) is the precursor of diterpenes and diterpenoids in their biosynthesis. By means of this linear 20-carbon isoprenyldiphosphate precursor, diterpene synthases generate diterpenes through a series of electrophilic cyclizations and/or rearrangements, starting with a carbocation formation and often ending by deprotonation [56].

**Scheme 5:**
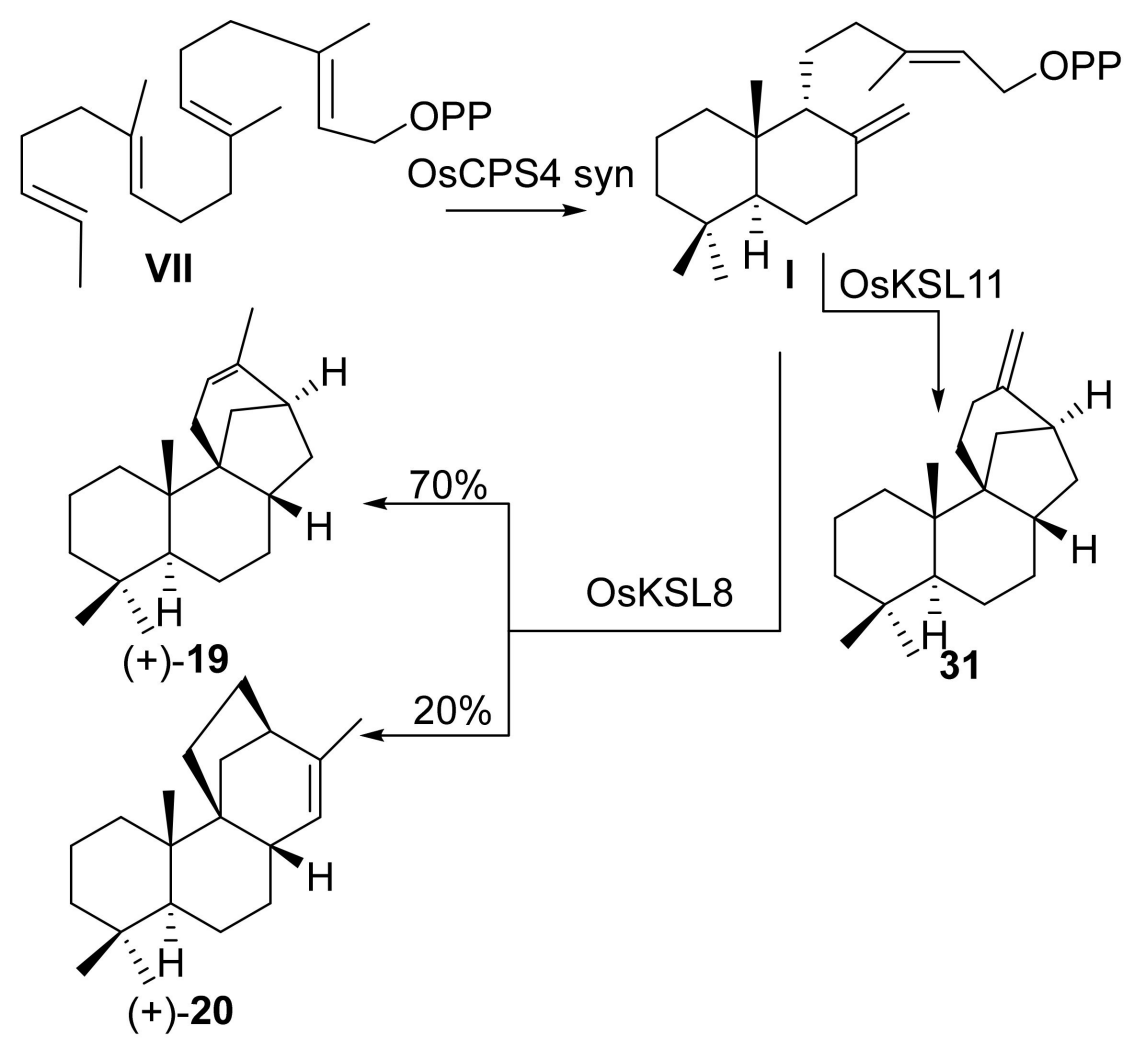
Proposed biosynthetic pathways for stemodane and stemarane diterpenoids in *Oryza sativa* L. (rice). OsCPS4, *syn*-copalyl diphosphate synthase; OsKSL8, *ent*-kaurene synthase-like 8; OsKSL11, *ent*-kaurene synthase-like 11.

GGPP (**VII**) is converted into *syn*-CPP (**I**) by class II terpenoid cyclases (Scheme 5). Several class I terpenoid cyclases are responsible for the further cyclization of CPP stereoisomers. *Ent*-kaurene synthase-like 8 and 11 (OsKSL8 and OsKSL11) are closely related (88.6% amino acid identity) as well as their encoding genes (92.0% nucleotide identity) [57]. Both enzymes are *syn*-CPP specific (Scheme 5). Unlike other OsKSL that produce only a single diterpene, OsKSL8 is able to generate two different diterpenes from *syn*-CPP, namely (+)-stemod-12-ene (**19**) (~20%) and (+)-stemar-13-ene (**20**) (~70%). The enzymatic product of OsKSL11 is represented, on the contrary, almost entirely by stemod-13(17)-ene (**31**) [58]. The gene encoding for OsKSL11 has been first discovered in 2006 by R.J. Peters (Iowa State University), R.M. Coates (University of Illinois, Urbana) and colleagues in rice (*Oryza sativa*) [58]. Functional characterization of OsKSL11 showed that it was a *syn*-CPP specific *exo*-stemodene synthase (Scheme 5). This finding was surprising, first because the *OsKSL11* sequence was not present in the rice genome database, then because stemodane type diterpenoids had not been yet identified in rice [58]. Even today, despite extensive phytochemical investigations, such compounds have not been identified in *Oryza* species. It should be noted that almost all of these studies focused on leaf phytoalexins. The Authors hypothesized that rice stemodane diterpenes could be produced and accumulated in other organs and/or in response to different stimuli (e.g., viral infections). Genes and enzymes involved in the biosynthesis of stemodane diterpenes and diterpenoids have not yet been identified so far in *Stemodia* species.

## 5. Biological Activity

As reported by D.F. Austin (University of Arizona), in the folk medicine of the Dutch Antilles, an infusion of leafy branches of sea-lavender (*Lavandula*) and *S. maritima*, mixed with Epsom salts, is used by men against venereal diseases [59]. Plants of *Calceolaria* genus are used in Central and South America popular medicine as stomach tonics, bactericidal agents and sweeteners [60]. In the frame of a study on plants toxic to ruminants and equine in Rio Grande do Norte western and eastern Seridó, Riet-Correa and co-workers (Universidade Federal de Campina Grande, PB) reported that some farmers consider *S. maritima* (melosa), which grows in areas rich of mineral salts, toxic and responsible, inter alia, of aborts. Nevertheless, the Authors report that *S. maritima* has not been described as toxic, nor have experiments proven its toxicity [61].

Essential oils obtained by the Arriaga group from *S. maritima* leaves and stems, collected in the state of Ceará, showed larvicidal properties against the *Aedes aegypti* mosquito larvae, responsible for the transmission of yellow fever in Central and South America and in West Africa and a vector of dengue hemorrhagic fever [62]. However, the main compounds obtained by hydrodistillation from leaves and stems were β-caryophyllene, 14-hydroxy-9-*epi*-β-caryophyllene and caryophyllene oxide. This biological activity cannot, therefore, be attributed to the stemodane diterpenes.

Besides, in 2017, the University of Ceará group, headed in this case by M.M. Bezerra, also observed that *S. maritima* extracts decreased inflammation, oxidative stress and alveolar bone loss in an experimental periodontitis rat model. In the frame of this work “no signs of systemic illness, adverse pharmacological events or changes in behavior were observed throughout the experimental period” [63].

Recently a phytochemical analysis and the gastro-protective effects of *S. maritima* hexane extract were also described [64].

Evaluations of pure stemodane diterpenoids were also made. Because of the structural similarity to (+)-aphidicolin (**28**) [51,52] in 1992, Hufford and colleagues tested for HSV-1 activity the diterpenes (+)-**1**, (+)-**13**, (+)-**14**, (+)-**15** and (+)-**16** [10]. “The antiviral activities ranged from 30–200 mg/mL with a maximum of 77% plaque reduction for stemodinoside B (**15**)” [10]. Compound (+)-**13** [10,11] was also tested, among other compounds, by Luo and colleagues [11] for its antibacterial properties against *Shigella flexneri*, *S. dysenteriae*, *S. sonnei*, *Mycobacterium tuberculosis*, α-hemolytic *Streptococcus*, and *Streptococcus pneumoniae* by means of the dose-dependent paper-disk diffusion method. The results are collected in Table 2 relative to rifampicin as a positive control.

From the results above reported, it appears that (+)-**13** is only weakly active against *S. sonnei*, *M. tuberculosis*, α-hemolytic *Streptococcus* and *S. pneumoniae*.

In 2014, Arriaga and colleagues evaluated the antioxidant power of (+)-stemodin (**1**) but found no activity in this respect [6]. The same Brazilian group also evaluated the MIC of (+)-stemodin (**1**) and (+)-stemodinoside B (**15**) (Table 3) against a number of bacteria. The best results were obtained with (+)-stemodinoside B (**15**) against *Klebsiella pneumoniae* and *Listeria monocytogenes* [6].

Finally, the Fu lead group evaluated the cytotoxicity of (+)-trigoheterone A (**18**) against five cancer cell lines (HL-60, SMMC-7721, A549, MCF-7 and SW480). As a result, (+)-**18** exhibited significant inhibitory effects with half-maximal inhibitory concentration (IC_50_) values comparable to those of cisplatin (Table 4) [14].

The use of plants producing stemodane diterpenes and diterpenoids in folk medicine as remedies in the treatment of several disorders is well documented [59,60]. Recent studies, even on animal models, have confirmed interesting biological activities of extracts from stemodane diterpene-producing plants such as *Calceolaria* spp. and *S. maritima* [61,62,63,64]. The antimicrobial activity of individual compounds such as (+)-stemodinoside B (**15**) was also quantified by in vitro tests [6]. The biological activity of some stemodane diterpenoids was examined in various respects: (+)-stemodinoside B (**15**) showed antiviral activity with a maximum of 77% plaque reduction [10]; (+)-maristeminol (**13**) was found to be weakly active towards some bacterial strains [11]; the antioxidant power of (+)-stemodin (**1**) was evaluated, but no activity was recorded [6]; the MIC of (+)-stemodin (**1**) and (+)-stemodinoside B (**15**) was evaluated against a number of bacterial strains: the best results were displayed by (+)-**15** against *Klebsiella pneumoniae* and *Listeria monocytogenes* [6]; finally, the cytotoxicity of (+)-trigoheterone A (**18**) against some cancer cell lines was tested resulting in significant inhibitory effects with IC_50_ values comparable to those of *cis*-platin [14].

## 6. Biotransformations

**Figure 9:**
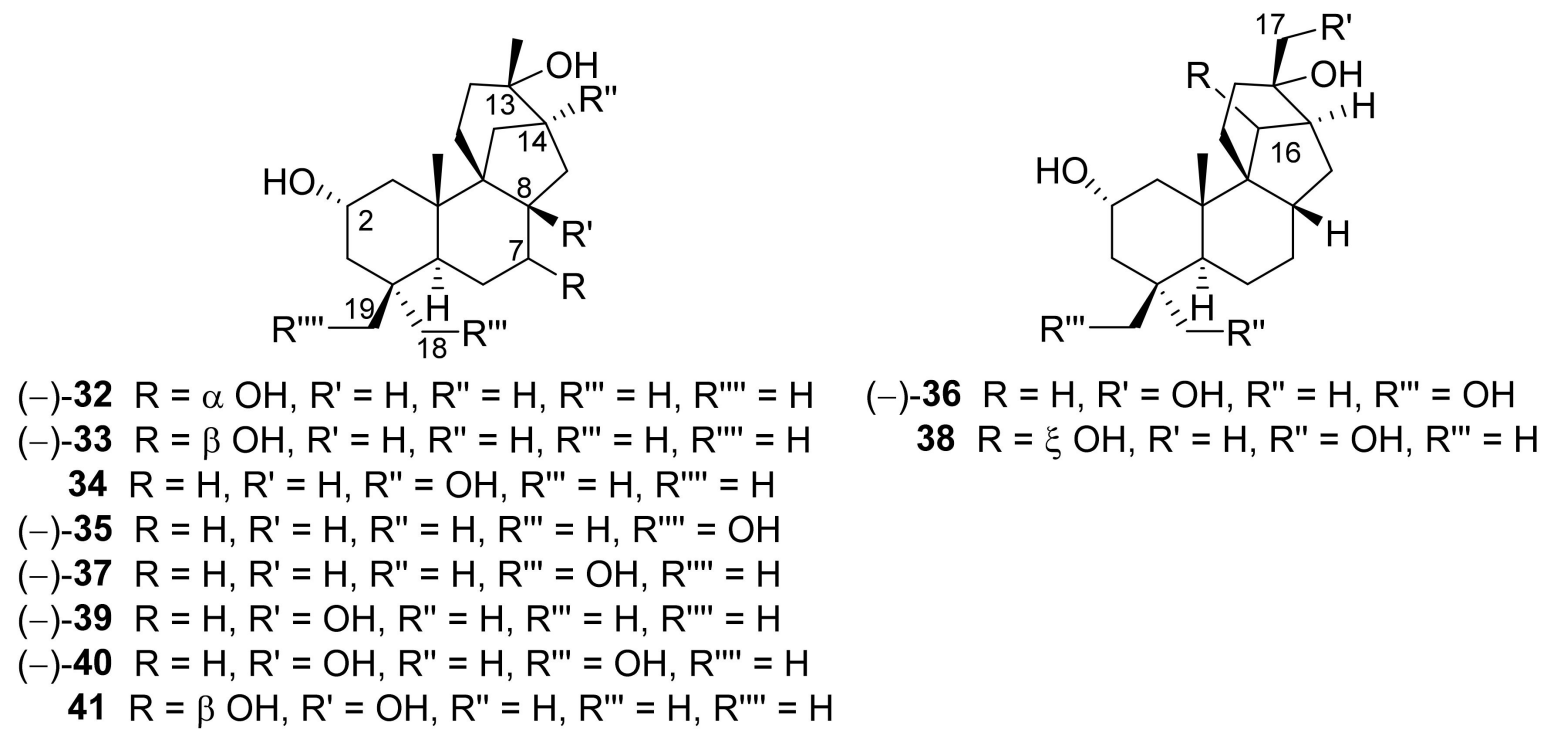
Stemodane diterpenoids biotransformation metabolites.

Aiming to produce new compounds, the obtaining of which by synthesis would have been too laborious, and to evaluate their biological activity, various research groups got involved in the biotransformation of stemodane diterpenoids. The work in this field of stemodane diterpenes biotransformations was pioneered by F.A. Badria and C.D. Hufford [65], who in 1991 screened a large number of microorganisms for their ability to transform (+)-stemodin (**1**). Among those *Cunninghamella echinulata* ATCC 9244 and *Polyangium cellulosum* ATCC 29610 were selected for preparative scale biotransformation because they could convert completely (+)-stemodin (**1**). The major metabolites were (−)-7α-hydroxystemodin (**32**), (−)-7β-hydroxystemodin (**33**), 14α-hydroxystemodin (**34**), (−)-19-hydroxystemodin (**35**) and (−)-17,19-dihydroxystemodin (**36**) (Table 5, entries 1, 2; Figure 9). Their identification was performed mainly by 2D-NMR.

The same Authors, in collaboration with W.T. Shier and M. Abou-Karam (University of Minnesota) and R.D. Rogers (Northern Illinois University) continued the above investigation screening more microorganisms and selecting *Rhizopus arrhizus* ATCC 11145 and *Streptomyces* sp. NRRL 5691 for preparative scale transformation because no (+)-stemodin (**1**) was detected in the fermentation broths [66]. The major metabolites formed were (−)-**32**, (−)-**33** ([α]_d_ in pyridine), (−)-18-hydroxystemodin (**37**), 16ξ,18-dihydroxystemodin (**38**), (−)-8β-hydroxystemodin (**39**), (−)-8β,18-dihydroxystemodin (**40**) and 7β,8β-dihydroxystemodin (**41**) (Table 5, entries 3, 4). Their identification was performed by 2D NMR and X-ray techniques. Metabolites (−)-**32**, (−)-**33**, **34**, (−)-**35,** (−)-**36,** (−)-**37**, **38,** (−)-**39,** (−)-**40** and **41** (Figure 9) were then tested to evaluate their antiviral and cytotoxic activity (vide infra).

**Figure 10:**
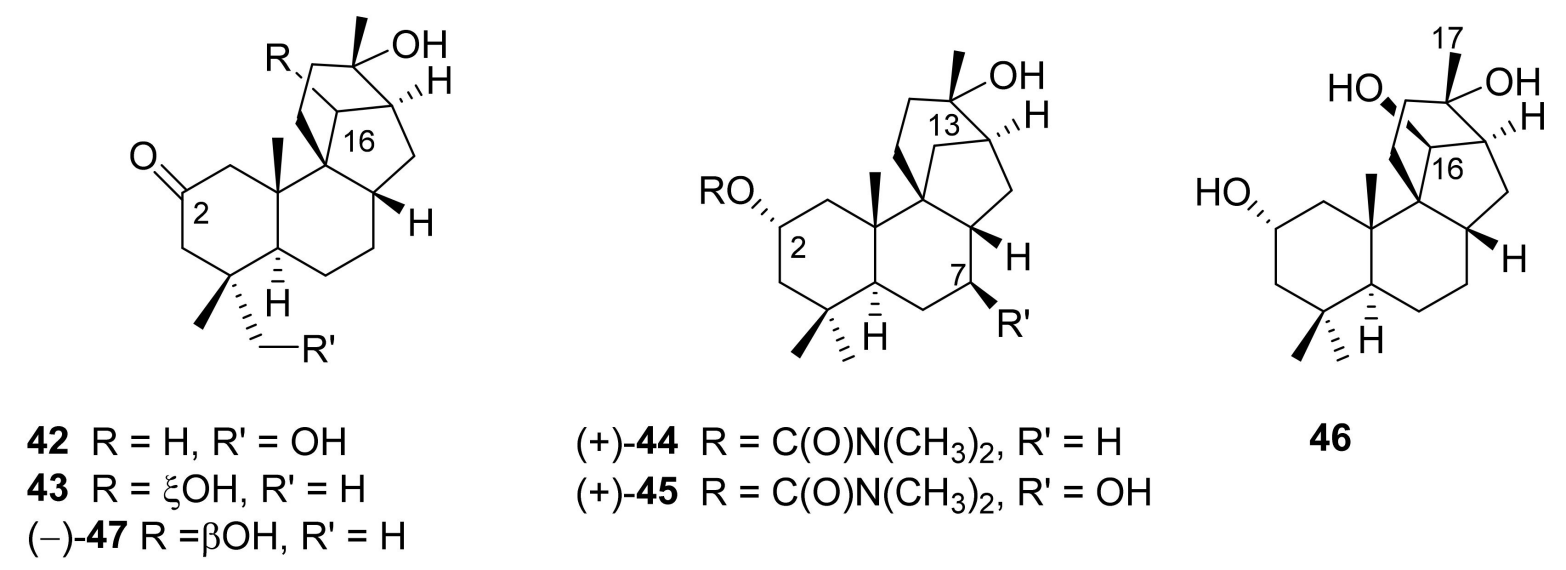
Stemodane diterpenoids biotransformation metabolites.

In 1994, P.B. Reese and M.R. Wilson (University of West Indies, Mona), in collaboration with J.R. Hanson and J.A. Takahashi (School of Molecular Sciences, University of Sussex), described the results of the incubation of (+)-stemodin (**1**) and (+)-stemodinone (**2**) with *Cephalosporium aphidicola* to afford (−)-7α-hydroxystemodin (**32**), (−)-7β-hydroxystemodin (**33**), (−)-19-hydroxystemodin (**35**), (−)-18-hydroxystemodin (**37**), (−)-8β-hydroxystemodin (**39**) (Figure 9), 18-hydroxystemodinone (**42**) and 16ξ-hydroxystemodinone (**43**), respectively (see page 17, Table 5, entries 5, 6; Figure 10) [67]. The structures of (−)-**32**, (−)-**33**, (−)-**35**, (−)-**37**, **42** and **43** were elucidated by means of IR, ^1^H- and ^13^C-NMR. Metabolites structures were established by Reese and colleagues by spectroscopic techniques also in the following studies.

Extensive work in the area of stemodane diterpenoids biotransformations was then continued in the following years by Reese and his group. In 2001, the hydroxylation at C(18) of (+)-stemodin (**1**) (Table 5, entry 7) and (+)-stemodinone (**2**) (Table 5, entry 8) by the action of *Beauveria bassiana* ATCC 7159, to give (−)-18-hydroxystemodin (**37**) and (+)-18-hydroxystemodinone (**42**) respectively was reported [68]. A number of derivatives of (+)-stemodin (**1**) were also prepared and incubated with cultures of *B. bassiana*.

Interestingly, (+)-2α-dimethylcarbamoxy-13-hydroxystemodane (**44**) was transformed by the microorganism into the (+)-7β-hydroxylated derivative **45** (Figure 10).

The Reese group also reported that, by the action of *Aspergillus niger* ATCC 9142, (+)-**1** is converted into metabolites (+)-maristeminol (**13**), (−)-7β-hydroxystemodin (**33**), 16β-hydroxystemodin (**46**), while (+)-stemodinone (**2**) was transformed into (+)-18-hydroxystemodinone (**42**) and (−)-16β-hydroxystemodinone (**47**) (see page 17, Table 5, entries 10, 11; Figure 10) [69].

Hypotheses about the hydroxylation of the above compounds by the involved enzymes in relation to the binding sites are also proposed [69].

**Figure 11:**
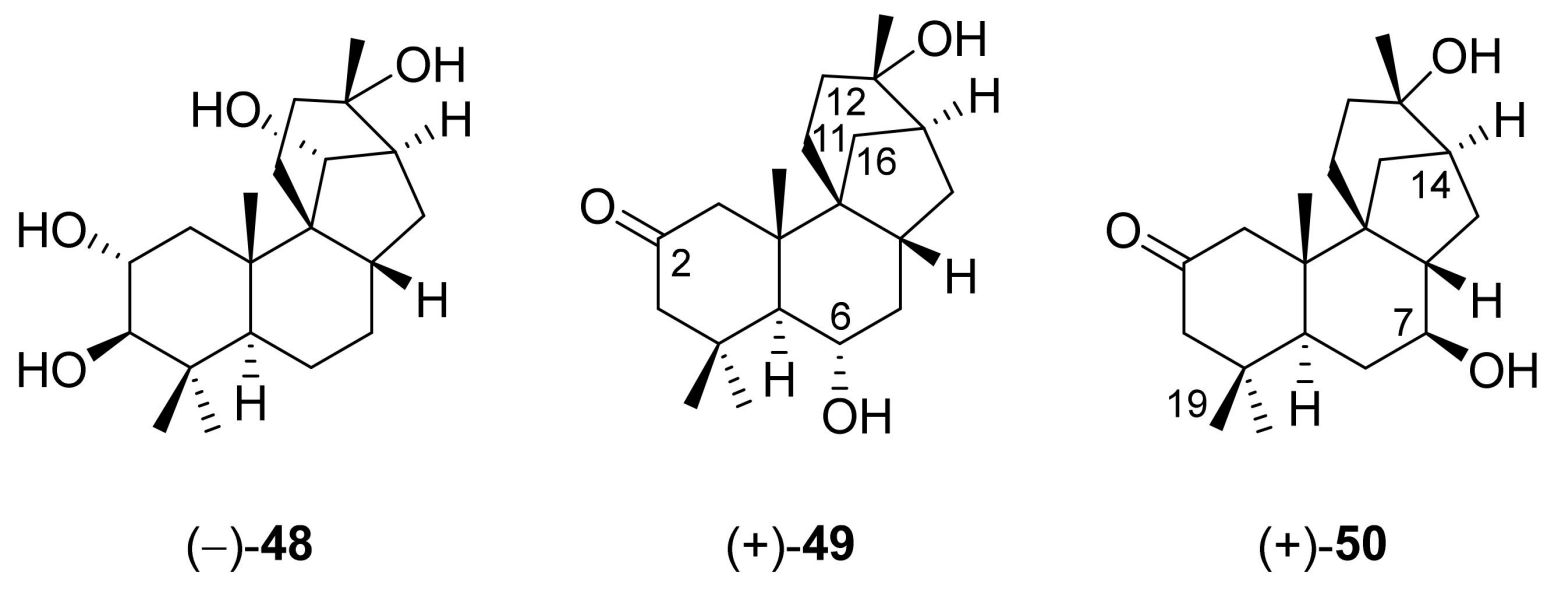
Stemodane diterpenoids biotransformation metabolites.

Later, Reese and his group reported, in collaboration with W.F. Reynolds (University of Toronto), the incubation of (+)-**1** with *Rhizopus oryzae* ATCC 11145, which gave (+)-7β-hydroxystemodin (**33**) ([α]_d_ in MeOH) and (−)-3β,16α-dihydroxystemodin (**48**) while the incubation of (+)-**2** gave (+)-6α-hydroxystemodinone (**49**) [12] (Figure 11).

**Figure 12:**
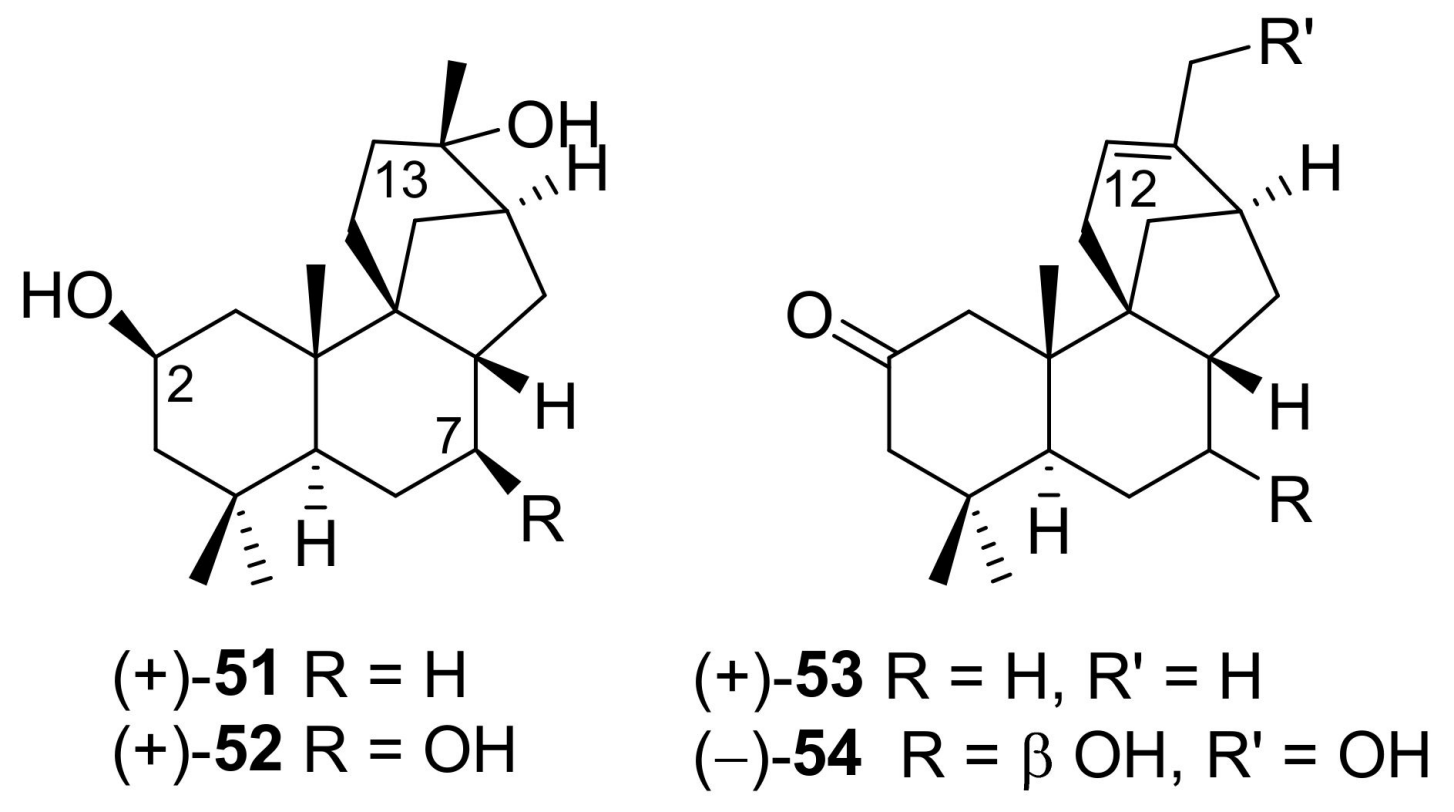
Stemodane diterpenoids biotransformation metabolites.

The bioconversion of (+)-2-*epi*-stemodin (**51**), obtained from (+)-**2** by NaBH_4_ reduction, gave (+)-2β,7β,13(*S*)—trihydroxystemodane (**52**) while the incubation of (+)-stemoden-12-en-2-one (**53**), obtained from (+)-**1** through a series of steps, gave (−)-7β,17-dihydroxystemod-12-en-2-one (**54**) [12] (Figure 12).

In order to investigate the importance of the C-2 and C-13 oxygen functions in the transformation of stemodin analogues by *Rhizopus oryzae* ATCC 11145, the same authors incubated with this microorganism a number of non-natural (+)-stemodin (**1**) derivatives epimeric at C(2) and/or C(13) and (+)-stemodinone (**2**) derivatives epimeric at C(13) [70].

**Figure 13:**
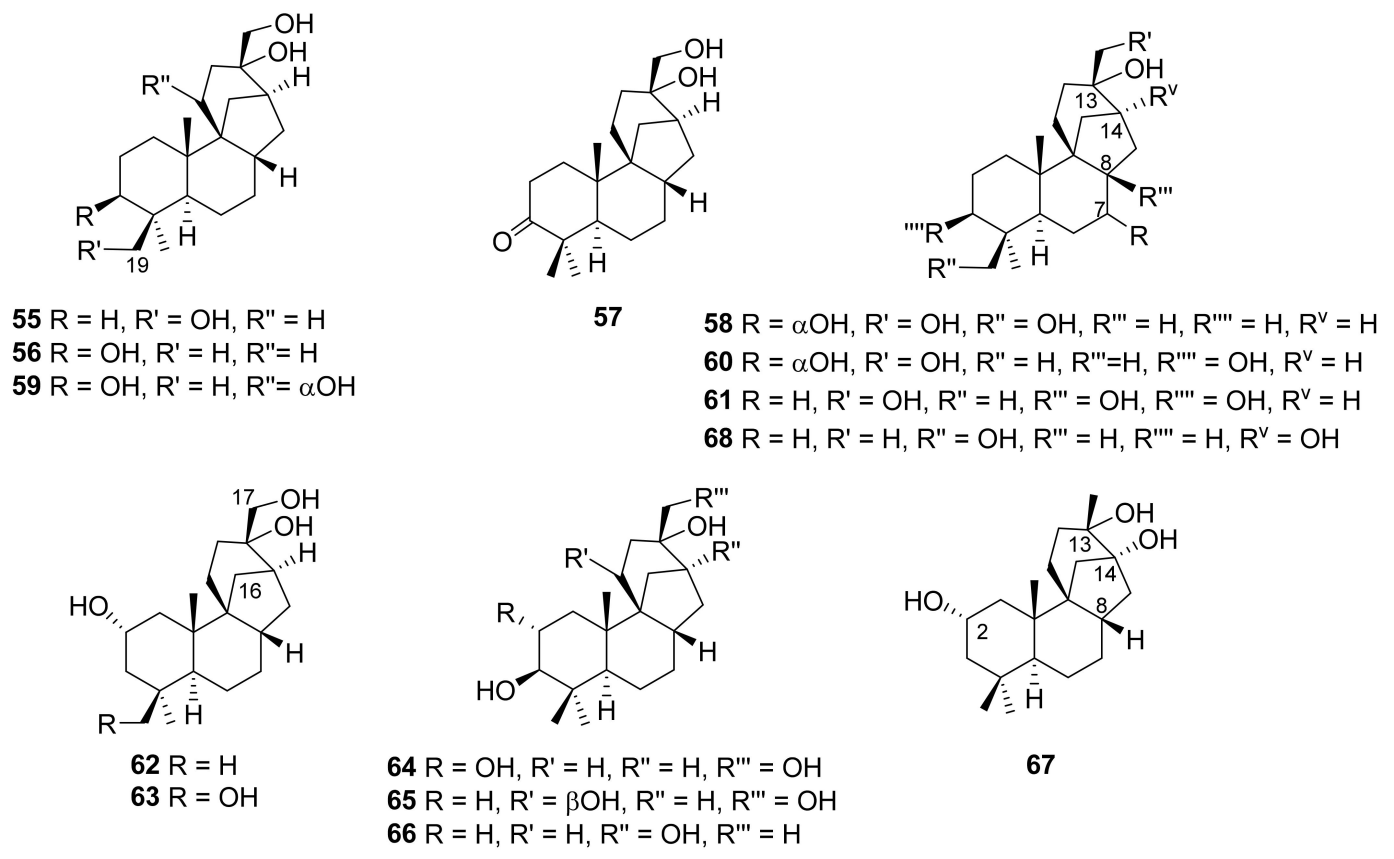
Stemodane diterpenoids biotransformation metabolites.

In 2004, B. M. Fraga (Instituto de Productos Naturales y Agrobiología, CSIC, La Laguna, Tenerife) and his group reported, in collaboration with R. Guillermo (Instituto Universitario de Bio-Orgánica “A. Gonzales”, Universidad de La Laguna, Tenerife) and the Garbarino group, the microbiological transformation of (+)-13α,17-dihydroxystemodane (**12**) by the action of the fungus *Mucor plumbeus* (Table 5, entries 16,17) to give 13α,17,19-trihydroxystemodane (**55**), 3β,13α,17-trihydroxystemodane (**56**), 3-oxo-13α,17-dihydroxy-stemodane **(57**), 7α,13α,17,19-tetrahydroxystemodane (**58**), 3β,11α,13α,17-tetrahydroxystemodane (**59**), 3β,7α,13α,17-tetrahydroxystemodane (**60**), 3β,8β,13α,17-tetrahydroxystemodane (**61**), 2α,13α,17-trihydroxystemodane (**62**), 2α,13α,17,19-tetrahydroxystemodane (**63**) 2α,3β,13α,17-tetrahydroxystemodane (**64**) and 3β,11β,13α,17-tetrahydroxystemodane (**65)** [71,72] (Figure 13).

The incubation of (−)-13α,14-dihydroxystemodane (**11**) gave 3β,13α,14-trihydroxystemodane (**66**), 2α,13α,14-trihydroxystemodane (**67**) and 13α,14,19-trihydroxystemodane (**68**) (Figure 13).

The regio- and stereochemistry of the hydroxylation was also discussed.

The structures of the above metabolites and those obtained in the following studies by the group were established by HRMS and ^1^H- and ^13^C-NMR [71,72].

In 2005, Reese and his group reported, in collaboration with Reynolds, the results obtained incubating (+)-stemodin (**1**) and (+)-stemodinone (**2**) with *Mucor plumbeus* ATCC 4740 (Table 5, entries 18, 19) [73]. The isolated metabolites obtained from (+)-**1** were 6β-hydroxystemodin (**69**), (+)-maristeminol (**13**), (−)-11β-hydroxystemodin (**70**) and (+)-14-hydroxystemodin (**34**), while (+)-**2** gave (+)-6α-hydroxystemodinone (**49**) and (+)-6α,12α-hydroxystemodinone (**71**). The biotransformations by *Whetzelinia sclerotiorum ATCC 18687* of (+)-stemodin **1** into 7β-hydroxystemodin (**33**) and (−)-11β-hydroxystemodin (**70**) and that of (+)-stemodinone (**2)** into (+)-7β-hydroxystemodinone (**50**) (Figure 11) were also reported (Table 5, entries 20, 21) [73].

**Figure 14:**
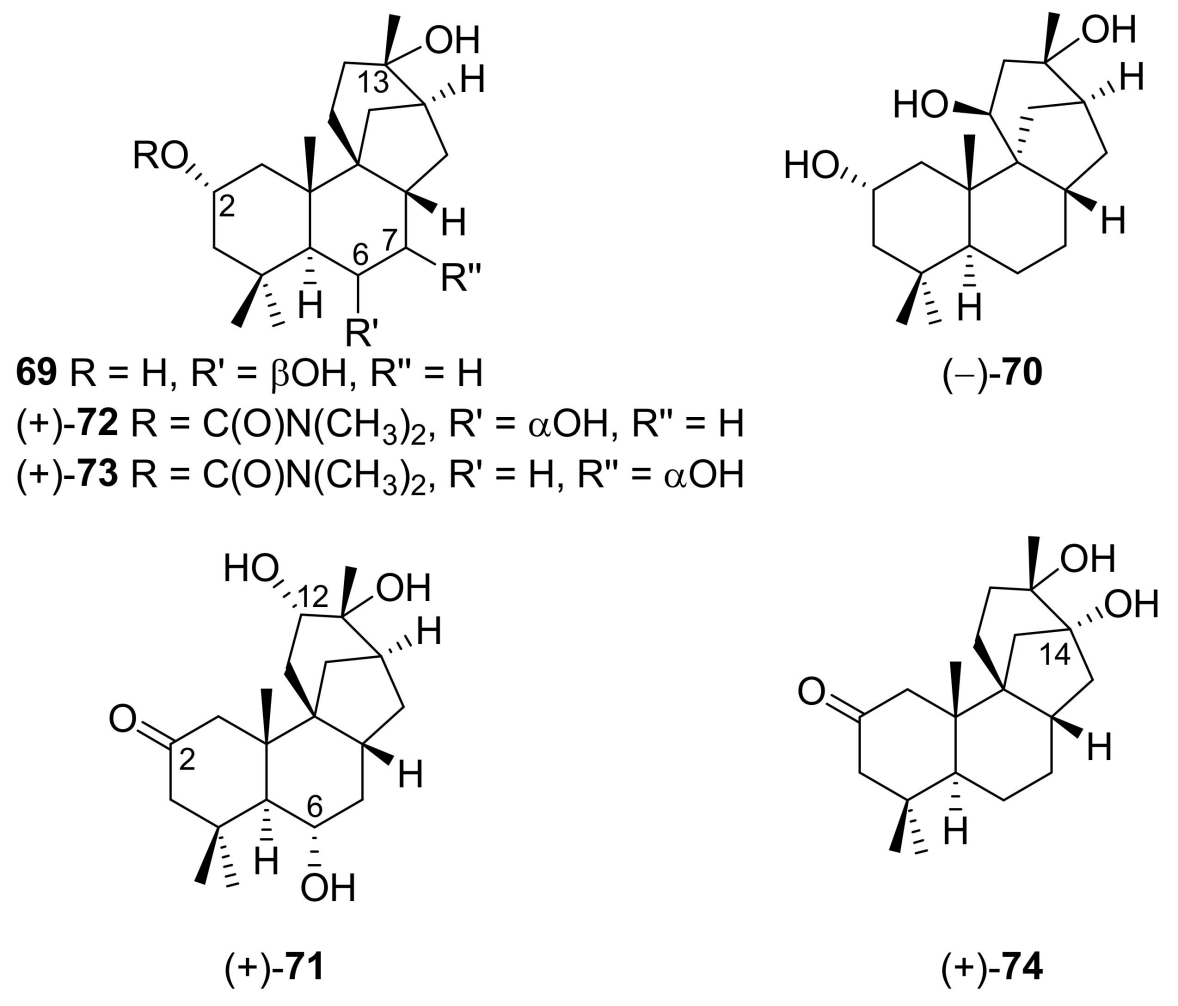
Stemodane diterpenoids biotransformation metabolites.

The same authors reported the bioconversion of (+)-stemodin (**1**) by *Cunninghamella echinulata var. elegans* (Table 5, entries 22, 23, 24) into (−)-7α-hydroxystemodin (**32**), (−)-7β-hydroxystemodin (**33**) and (+)-maristeminol (**13**) [74]. (+)-2α-(*N*,*N*-dimethylcarbamoxy)-13-hydroxystemodane (**44**) gave (−)-2α-(*N*,*N*-dimethylcarbamoxy)-6α,13-dihydroxystemodane (**72**) and (+)-2α-(*N*,*N*-dimethylcarbamoxy)-7α,13-dihydroxystemodane (**73**). The same microorganism converted (+)-stemodinone (**2**) into (+)-14-hydroxystemodinone (**74**) (Figure 14) and (+)-7β-hydroxystemodinone (**50**) (Figure 11).

By the action of *Phanerochaete chrysosporium* (Table 5, entries 25, 26, 27) (+)-stemodin (**1**) was transformed into (−)-7β-hydroxystemodin (**33**), (+)-maristeminol (**13**) and (−)-11β-hydroxystemodin (**70**) while (+)-stemodinone (**2**) was converted into 18-hydroxystemodinone (**42**) [67,74] (Figure 14).

**Figure 15:**
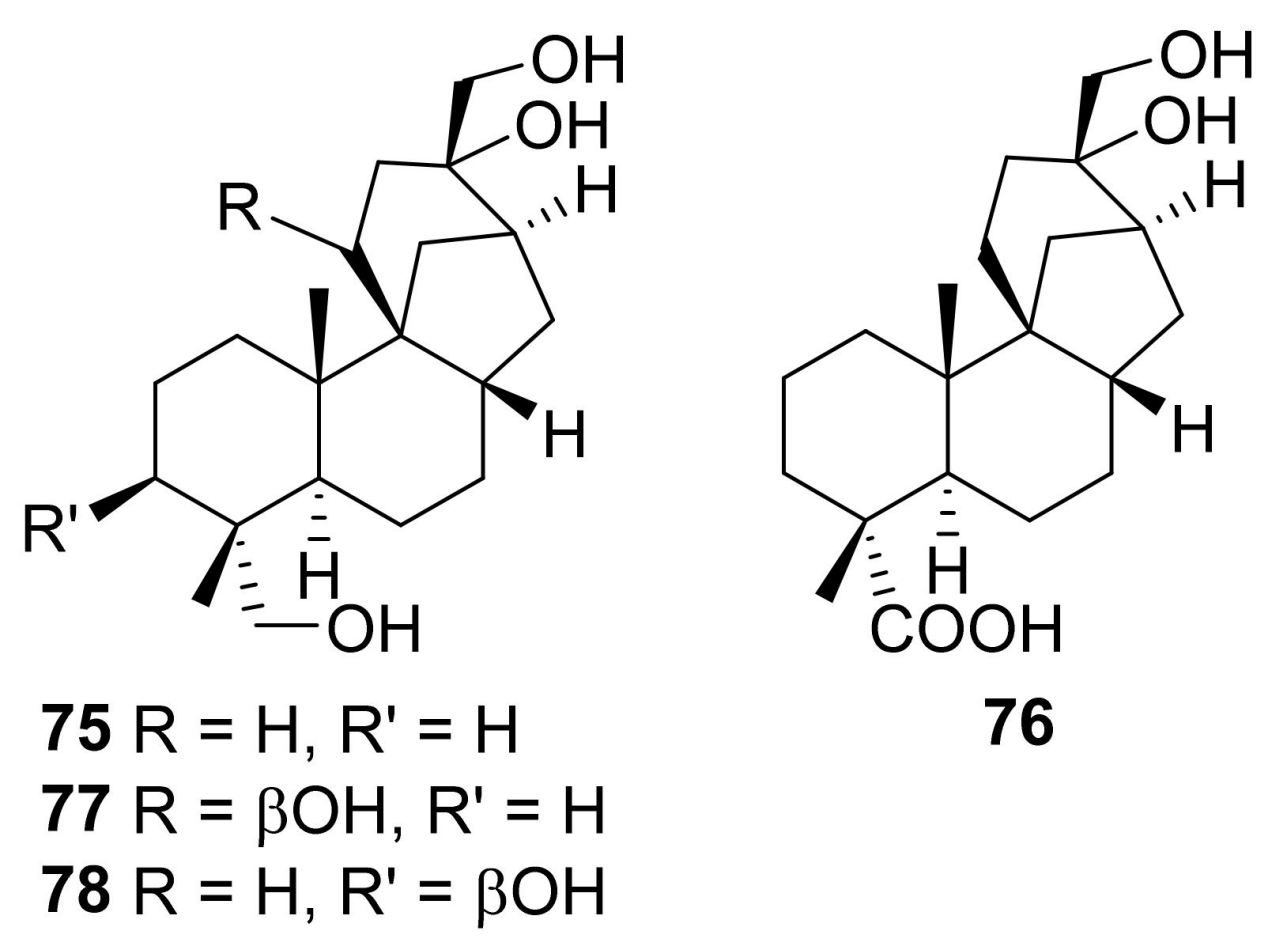
Stemodane diterpenoids biotransformation metabolites.

In 2012, Fraga and his group reported, in collaboration with Guillermo, Garbarino and M. C. Chamy (Universidad Andrés Bello, Viña del Mar) the biotransformation of 13α,17-trihydroxystemodane (**12**) by the action of the fungus *Cephalosporium aphidicola* (Table 5, entry 28) to give 13α,17,18-trihydroxystemodane (**75**), 3β,13α,17-trihydroxystemodane (**56**), 13α,17-dihydroxystemodan-18-oic acid (**76**), 3β,11β,13α,17-tetrahydroxystemodane (**65**), 11β,13α,17,18-tetrahydroxystemodane (**77**) and 3β,13α,17,18-tetrahydroxystemodane (**78**) [75] (Figure 15).

Hypotheses on xenobiotic biotransformations vs. biosynthetically-directed transformations in relation to the hydroxylated sites and the role of nearby hydroxyls were also put forward.

**Scheme 6:**
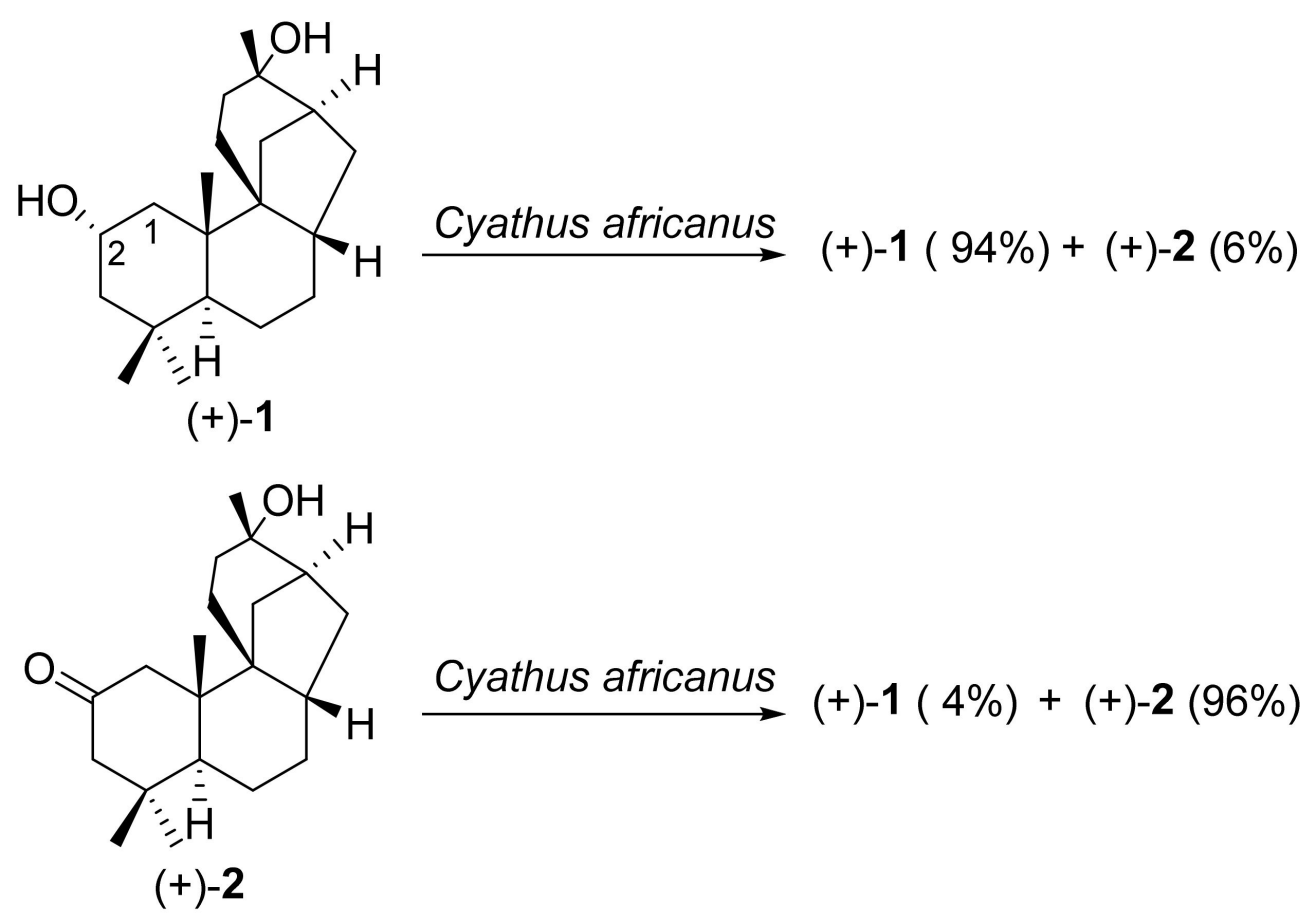
Incubation of (+)-stemodin (**1**) and (+)-stemodinone (**2**) with *Cyathus africanus*.

Finally, very interesting observations were made by Reese and his group in collaboration with Reynolds. It was found that the incubation of (+)-stemodin (**1**) with *Cyathus africanus* leads, though in low yield, to (+)-stemodinone (**2**), while that of (+)-stemodinone (**2**) leads to (+)-stemodin (**1**) [76] (Scheme 6).

**Scheme 7:**
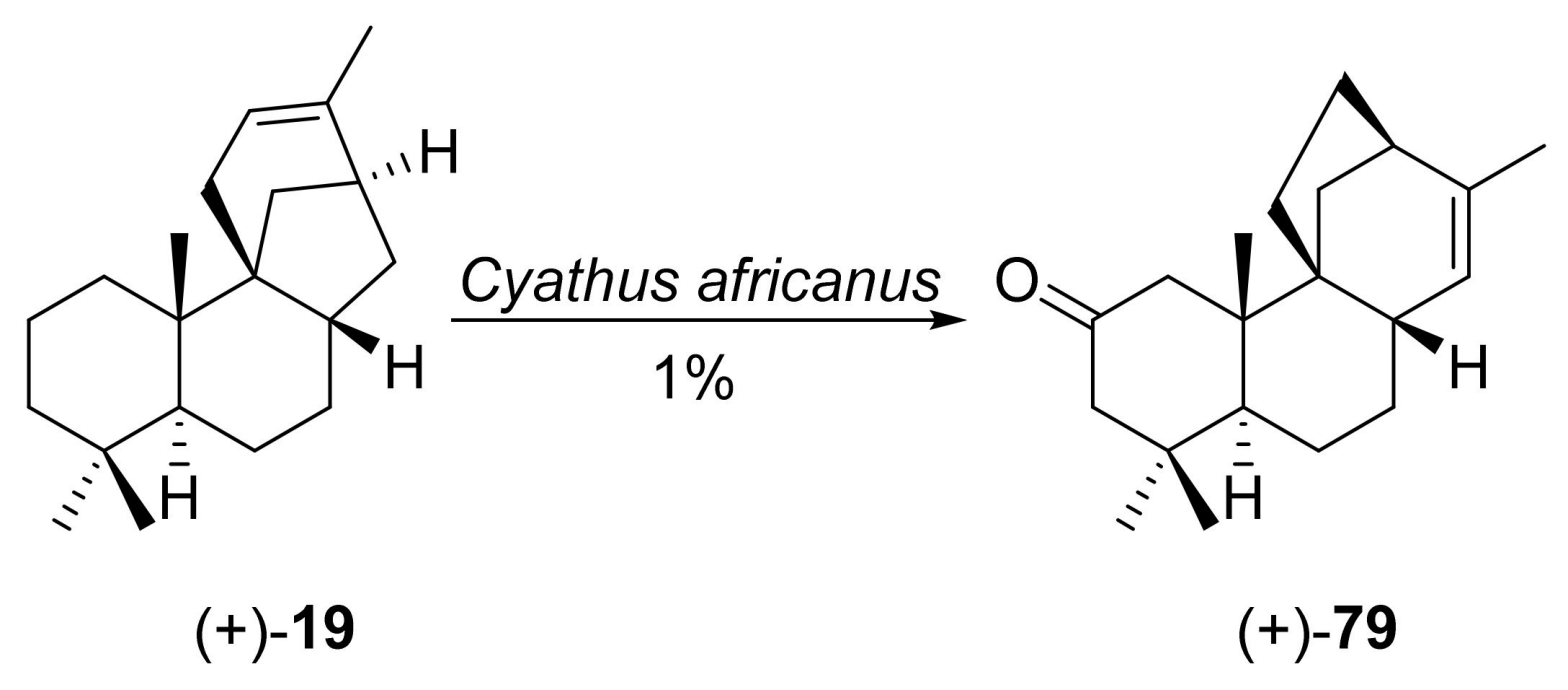
Conversion of **(+)-**12-stemodene (**19**) into (+)-2-oxo-13-stemarene (**79**) by the fungus *Cyathus africanus* [76].

In order to avoid the redox process and to have better substrates for hydroxylation, (+)-stemodinone (**2**) was deoxygenated to (+)-2-deoxystemodinone (**6**) and later dehydrated to (**19**). It was then found that (+)-**6** did not produce any metabolites by the action of the fungus, while (+)-12-stemodene (**19**), under the same conditions, the biotransformation by the fungus *Cyathus africanus* wass carried out, was not only oxidized at C(2) but the skeleton was rearranged to the stemarene one giving (+)-2-oxo-13-stemarene (**79**) [76] (Scheme 7). Nevertheless, the yield of this process was very low (1%).

**Table 5:** Metabolites obtained by the action of various microorganisms on stemodane diterpenoids and derivatives.

Entry	Starting Material	Microorganism	Metabolites	Reference/Year
1	(+)**-1**	*Cunninghamella echinulata*	(−)-**32** (−)-**33** **34**	[65]/1991
2	(+)-**1**	*Polyangium cellulosum*	(−)-**35** (−)-**36**	[65]/1991
3	(+)-**1**	*Rhizopus arrhizus* ATCC 11145	(−)-**32** (−)-**33** (−)-**37** **38**	[66]/1991
4	(+)-**1**	*Streptomyces* sp. NRRL 5691	(−)-**39** (−)-**40** **41**	[66]/1991
5	(+)-**1**	*Cephalosporium aphidicola*	(−)-**32** (−)-**33** (−)-**35** (−)-**37**	[67]/1994
6	(+)-**2**	*Cephalosporium aphidicola*	**42** **43**	[67]/1994
7	(+)-**1**	*Beauveria bassiana* ATCC 7159	(−)-**37**	[68]/2001
8	(+)-**2**	*Beauveria bassiana* ATCC 7159	(+)-**42**	[68]/2001
9	(+)-**44**	*Beauveria bassiana* ATCC 7159	(+)-**45**	[68]/2001
10	(+)-**1**	*Aspergillus niger* ATCC 9142	(+)-**13** (−)-**33** **46**	[69]/2002
11	(+)-**2**	*Aspergillus niger* ATCC 9142	(+)-**42** (−)-**47**	[69]/2002
12	(+)-**1**	*Rhizopus oryzae ATCC 11145*	(+)-**33** (−)-**48**	[12]/2004
13	(+)-**2**	*Rhizopus oryzae ATCC 11145*	(+)-**49** (+)-**50**	[12]/2004
14	(+)-**51**	*Rhizopus oryzae ATCC 11145*	(+)-**52**	[12]/2004
15	(+)-**53**	*Rhizopus oryzae ATCC 11145*	(−)-**54**	[12]/2004
16	(+)-**12**	*Mucor plumbeus*	**55** **56** **57** **58** **59** **60** **61** **62** **63** **64** **65**	[72]/2004
17	(−)-**11**	*Mucor plumbeus*	**66** **67** **68**	[72]/2004
18	(+)-**1**	*Mucor plumbeus*	(+)-**13** (+)-**34** **69** (−)-**70**	[73]/2005
19	(+)-**2**	*Mucor plumbeus*	(+)-**49** (+)-**71**	[73]/2005
20	(+)-**1**	*Whetzelinia sclerotiorum ATCC. 18687*	**33** (−)-**70**	[73]/2005
21	(+)-**2**	*Whetzelinia sclerotiorum ATCC. 18687*	(+)-**50**	[73]/2005
22	(+)-**1**	*Cunninghamella echinulata var. elegans*	(+)-**13** **32** **33**	[74]/2006
23	(+)-**44**	*Cunninghamella echinulata var. elegans*	(−)-**72** (+)-**73**	[74]/2006
24	(+)-**2**	*Cunninghamella echinulata var. elegans*	(+)-**50** (+)-**74**	[74]/2006
25	(+)-**1**	*Phanerochaete chrysosporium*	(+)-**13** **33** (−)-**70**	[74]/2006
26	(+)-**44**	*Phanerochaete chrysosporium*	untransformed	[74]/2006
27	(+)-**2**	*Phanerochaete chrysosporium*	**42**	[74]/2006
28	**12**	*Cephalosporium aphidicola*	**56** **65** **75** **76** **77** **78**	[75]/2012
29	(+)-**1**	*Cyathus africanus*	(+)-**2**	[76]/2012
30	(+)-**2**	*Cyathus africanus*	(+)-**1**	[76]/2012
31	(+)-**6**	*Cyathus africanus*	no metabolites	[76]/2012
32	(+)-**19**	*Cyathus africanus*	(+)-**79**	[76]/2012

From Table 5, it appears that the microorganisms tested are capable of hydroxylating all suitable C-atoms of the stemodane skeleton, but C(1), C(5), C(12), C(15) and C(20). It also appears that they hydroxylate more than a C-atom. Their regioselectivity is different; as an example, *Cunninghamella echinulata* hydroxylates (+)-stemodin (**1**) at C(7) and C(14) (entry 1) while *Polyangium cellulosum* at C(19) and C(17) (entry 2).

*Beauveria bassiana* ATCC 7159 represents an exception in that (+)-stemodin (**1**) and (+)-stemodinone (**2**) are regioselectively transformed in the corresponding 18-hydroxyderivatives **37** and **42,** respectively (entry 7 and 8). Besides, the same microorganism transforms (+)-2α-dimethylcarbamoxy-13-hydroxystemodane (**44**) into the corresponding 7β-hydroxy derivative **45** (entry 9).

In recent years, numerous derivatives of stemodane diterpenes have been obtained by means of biotransformation and some of them have shown remarkable biological activities, highlighting their potential as active ingredients for pharmaceutical, agricultural and industrial applications. In our opinion, the research activity in this area is currently underdeveloped and it would be beneficial to improve efforts for the discovery of new molecules and for preclinical and clinical experimentation, mainly focusing on the development of new antiviral, antibacterial and anticancer drugs.

Biotransformations of some stemodane diterpenoids have been reviewed [77,78,79,80,81,82].

## 7. Stemodane Diterpenoid Metabolites and Derivatives Biological Activity

Metabolites **32**–**41**, obtained as described above by microorganism transformation, were tested by Hufford and colleagues for antiviral and cytotoxicity activities using (+)-aphidicolin (**28**) as a positive control. The compounds were tested against Herpes simplex type 1 (HS-1). Each compound exhibits some cytotoxicity. Compound **39** had the highest activity being able to reduce the number of plaques by 93% at a concentration of 0.1 mg/mL. Compounds (−)-**33**, (−)-**36**, (−)-**40** and **41** did not show any reduction in the number of plaques at the same concentration [66]. (+)-Stemodin (**1**) and several analogues exhibited weak antiviral activity (Table 6).

Besides, the (+)-stemodin (**1**) analogues/derivatives (+)-**80**, (+)-**81**, (−)-**82**, (+)-**83**, (+)-**84**, (+)-**85**, (−)-**86**, (+)-**87** and (+)-stemod-12-en-2-one (**53**) were prepared from (+)-stemodin (**1**). The structures of (+)-**80**, (+)-**81**, (−)-**82**, (+)-**83**, (+)-**84**, (+)-**85**, (−)-**86** and (+)-**87** were established by spectroscopic techniques. Single crystal X-ray analyses confirmed the proposed structures for (+)-**81** and (−)-**82**.

**Figure 16:**
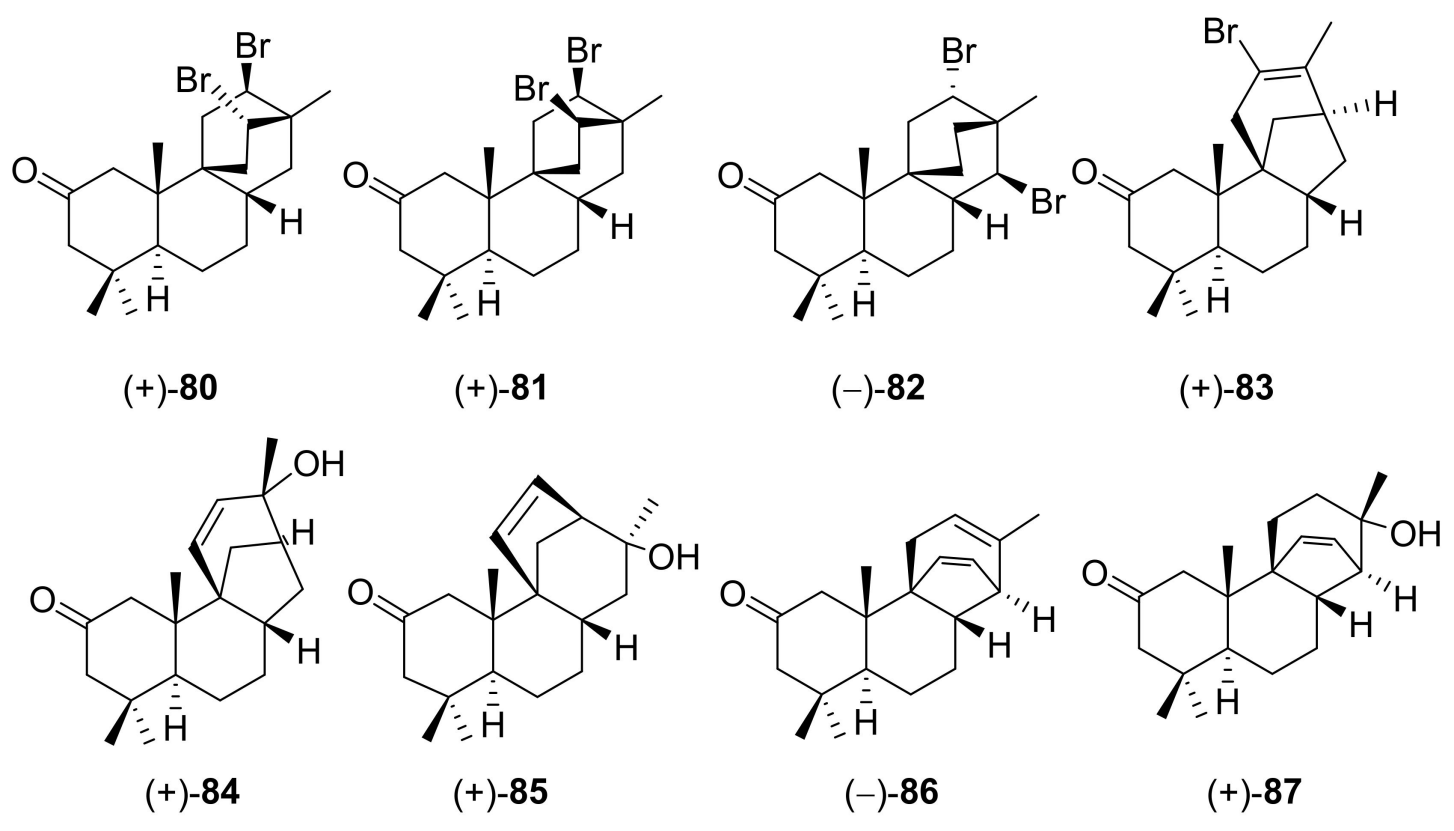
(+)-Stemodin (**1**) analogues and derivatives tested.

Compounds (+)-**80**, (+)-**81**, (−)-**82**, (+)-**83**, (+)-**84**, (+)-**85**, (−)-**86** and (+)-**87**, (+)-stemod-12-en-2-one (**53**) were tested, along with (+)-stemodin (**1**) and (+)-stemodinone (**2**) by Reese and colleagues to evaluate their lipid peroxidation (LPO) activity, cyclooxygenase enzyme-1 (COX-1) and -2 (COX-2) activity, and tumour cell proliferation inhibitory activity [83] (Figure 16).

**Figure 17:**
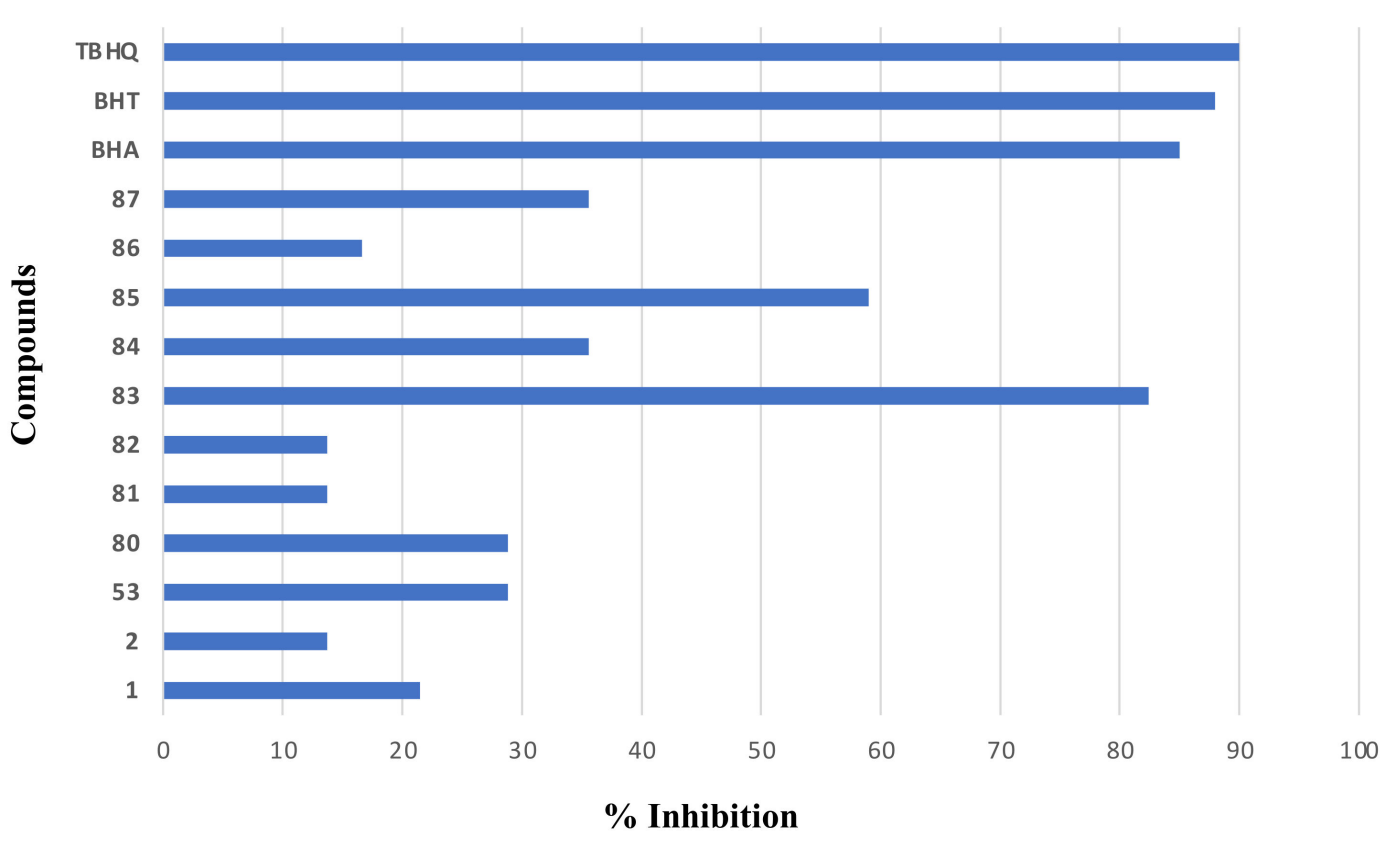
Lipid peroxidation inhibitory activity of (+)-stemodin (**1**) analogues and positive controls, BHA, BHT and TBHQ. Compounds were tested at 25 μg/mL. Positive controls were tested at 10 μM [83].

In the lipid peroxidation inhibitory assay (Figure 17) compounds analogues/derivatives (+)-**83**, (+)-**84** and (+)-**85** exhibited prominent activity while that of (+)-**53**, (+)-**80** and (+)-**87** was slightly superior to that of (+)-stemodin (**1**).

**Figure 18:**
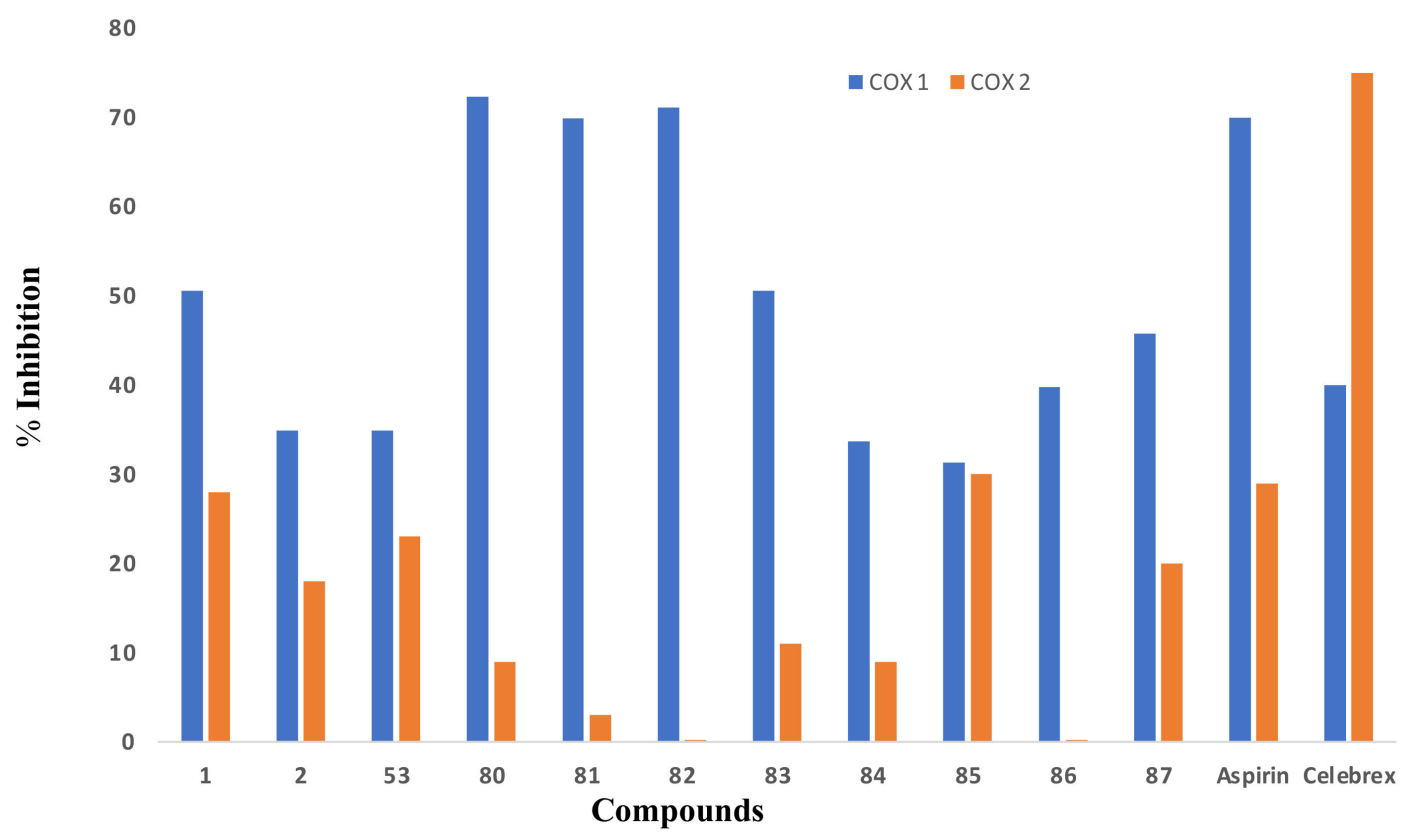
Cyclooxygenase enzyme-1 and -2 inhibitory activities of (+)-stemodin (**1**) analogues. Compounds were tested at 25 μg/mL. Positive controls Aspirin and Celebrex^TM^ were tested at 60 μM and 26 nM concentrations [83].

The inhibition of dibrominated analogues (+)-**80**, (+)-**81** and (−)-**82** was higher than that of stemodin (**1**), while those of non-brominated derivatives (+)-**84**, (+)-**85**, (−)-**86** and (+)-**87** resulted in being lower. Besides, all derivatives showed higher COX-1 enzyme inhibition than COX-2 (Figure 18).

**Figure 19:**
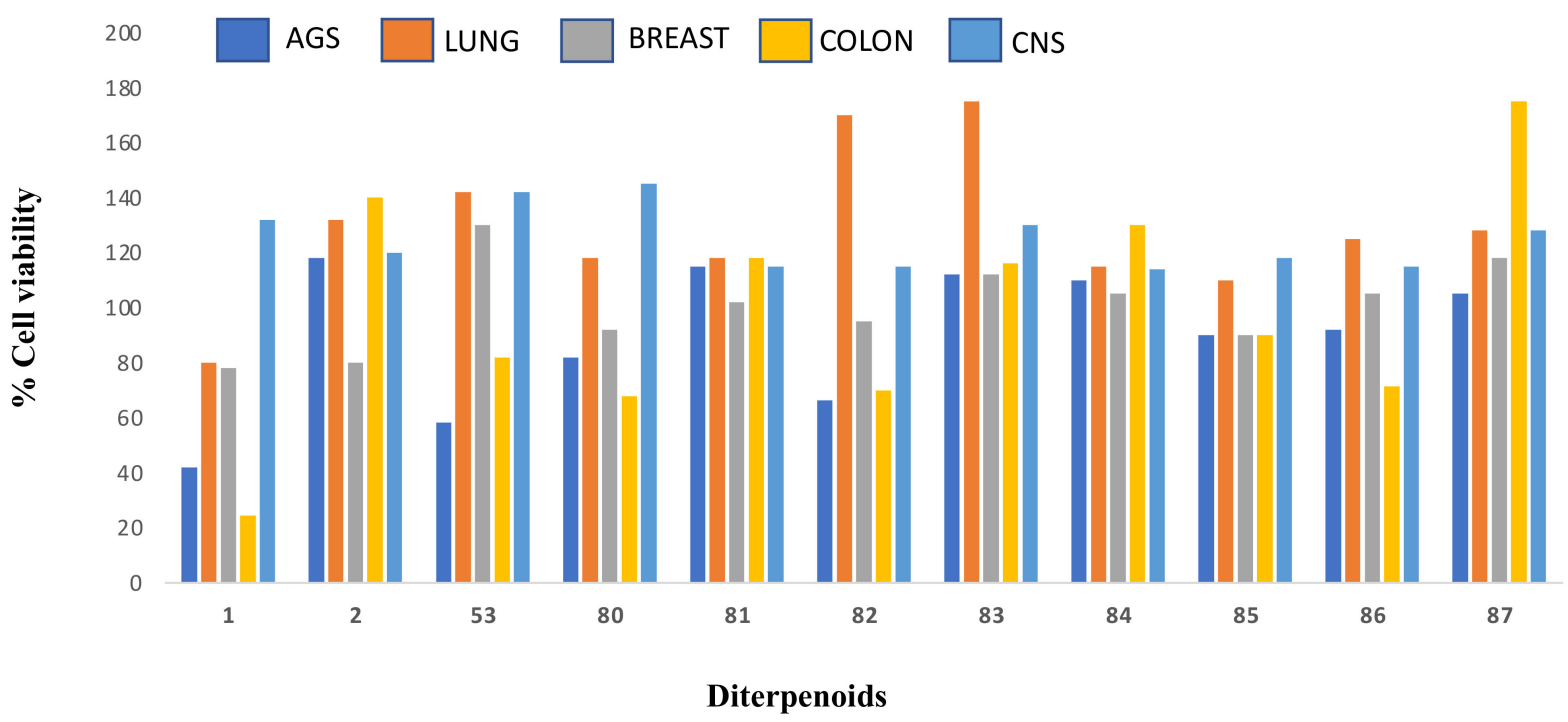
(+)-Stemodin (**1**) analogues cell viability against human gastric (AGS), NCI-H460 (lung), MCF-7 (breast), HCT-116 (colon) and SF-268 (central nervous system, CNS) cancer cell lines. Compounds were tested at 25 μg/mL. Samples were prepared in DMSO (0.2%) supplemented with RPMI-1640 medium [83].

As it can be seen, only a few of the above compounds limited the growth of some human tumor cell lines. On the contrary, most of them induced proliferation of such cells (Figure 19) [83].

Thus, among metabolites **32**–**41**, compound (−)-**39** showed the highest cytotoxicity. Weak antiviral activity was displayed by (+)-stemodin (**1**) and (−)-**32**, **34**, (−)-**37** and (−)-**39**. As far as analogues/derivatives **80**–**87** are concerned, in the LPO three compounds showed prominent activity; besides all derivatives displayed higher COX-1 enzyme inhibition than COX-2; finally, only few of the latter compounds limited the growth of some human tumour cell lines while most of them induced the cell proliferation.

## 8. Rearrangements

**Scheme 8:**
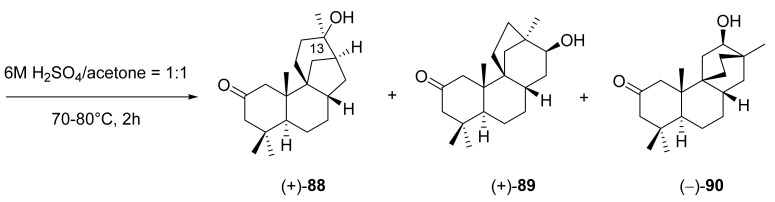
Rearrangement of (+)-**2** to (+)-**88**, (+)-**89** and (−)-**90** [84].

Solvolytic rearrangement of (+)-stemodinone (**2**) gave (+)-13-*epi*-stemodinone (**88**) and the tetracyclic compounds (+)-**89** and (−)-**90** [84] (Scheme 8). A mechanistic hypothesis for the formation of (+)-(**88**), (+)-**89** and (−)-**90** was given.

## 9. Conclusions

In this review, the work which involved various research groups for about forty-five years on stemodane diterpenes and diterpenoids structure elucidation, biogenesis, biosynthesis, biological activity and biotransformations is described.

The efforts which led to the structure elucidation of tetracyclic diterpenoids with a new skeleton, at a time in which ^13^C-NMR and advanced analytical techniques were not available yet, were described.

The probable biogenesis of stemodane diterpenes, as well as the work which allowed identification of the enzymes responsible for stemodane diterpenes biosynthesis was reviewed.

The biological activity of (+)-stemodin (**1**), congeners, metabolites and derivatives was also taken into account, as well as the biotransformations operated by a number of microorganisms, which permit to hydroxylate most of the stemodane nucleus C-atoms, allowing easier access, compared to total synthesis, to hydroxylated derivatives.

This class of structurally intriguing compounds was also the target of a large number of syntheses. Owing to the vastness of this aspect of stemodane diterpenoids chemistry, they will be considered at another time.

## Figures and Tables

**Table 1:** Summary table for compounds isolated from *S. maritima, S. chilensis,* *Homalomena* *occulta, Trigonostemon* *heterophyllus* (n.r. = not reported; n.a.E. = not available in English).

Entry	Plant	Collection Location	Collection Period	Plant Part Extracted	Extraction Method	Stemodane Isolated Compounds	Reference/Year
1	*S. maritima*	Palisadoes peninsula (Jamaica)	n.r.	leaves	n.r.	(+)-**1**, (+)-**2**	[1]/1973
2	*S. maritima*	Palisadoes peninsula (Jamaica)	n.r.	leaves	n.r.	(+)-**6**	[3]/1975
3	*S. maritima*	Curaçao	n.r.	above ground portion	available	(+)-**1**, (+)-**2**, (+)-**3**, (+)-**8**	[2]/1976
4	*S. chilensis*	Littoral zone Concón, Valparaiso Region (Chile)	December	aerial parts	available	(+)-**6**, (+)-**9**, (+)-**10**, (−)-**11**, (+)-**12**	[8]/1991
5	*S. maritima*	Curaçao	n.r.	whole plant	available	(+)-**13**, (+)-**14**, (+)-**15**, (+)-**16**	[10]/1992
6	*S. maritima*	n.r.	n.r.	n.r.	available	**1**, **14**, **15**, (−)-**17**	[12]/2004
7	*H. occulta*	Xishuangbanna (Yunnan Province, P. R. China)	Jul.	aerial parts	available	(+)-**13**	[11]/2007
8	*S. maritima*	Flexeiras-CE (Brasil)	Flowering stage	fresh stems	available	(+)-**1**	[13]/2010
9	*S. maritima*	Flexeiras-CE (Brasil)	Flowering stage	leaves and roots	available	(+)-**1**, (+)-**4**, (+)-**15**	[6]/2014
10	*T. heterophyllus*	n.a.E.	n.a.E.	stems and leaves	n.a.E.	(+)-**18**	[14]/2018

**Table 2:** Antibacterial Activities of (+)-**13** towards different bacterial strains. All values were determined by the paper-disk method and are expressed in terms of minimal inhibitory amount (MIA; in μg/disk) [11].

Compound	*Shigella* *flexneri*	*Shigella* *dysenteriae*	*Shigella* *sonnei*	*Mycobacterium* *tuberculosis*	α-Hemolytic *streptococcus*	*Streptococcus pneumoniae*
**(+)-13**	n.a. ^(a)^	n.a.	300	400	350	250
Rifampicin ^(b)^	1	1	1	1	1	1

^(a)^ Not active. ^(b)^ Positive control.

**Table 3:** Values of the minimal inhibitory concentration (MIC) (μg/mL) of (+)-stemodin (**1**) and (+)-stemodinoside B (**15**) [6]. ND = not determined.

Bacteria	(+)-1	(+)-15
*Staphylococcus aureus* (ATCC 12692)	512	≥1024
*Staphylococcus aureus* (ATCC 6538)	ND	512
*Staphylococcus aureus* (ATCC 358)	512	ND
*Staphylococcus aureus* (ATCC 12624)	ND	≥1024
*Pseudomonas aeruginosa* (ATCC 15442)	512	512
*Bacillus cereus* (ATCC 33018)	512	≥1024
*Escherichia coli* (ATCC 27)	512	≥1024
*Escherichia coli* (ATCC 25922)	≥1024	512
*Aeromonas carveae* (ATCC 15468)	ND	≥1024
*Klebsiella pneumoniae* (ATCC 10031)	ND	256
*Shigella flexneri* (ATC 12022)	ND	512
*Vibrio cholerae* (ATCC 15748)	ND	≥1024
*Listeria monocytogenes* (ATCC 7644)	ND	256

**Table 4:** Values of IC_50_/μmol^.^ L^−1^of (+)-trigoheterone A (**18**) [14].

IC_50_/μmol·L^−1^					
Compound	HL-60	SMMC-7721	A-549	MCF-7	SW480
(+)-**18**	0.98 ±0.07	1.87 ± 0.10	2.05 ± 0.09	0.86 ± 0.06	1.69 ± 0.08
cisplatin	1.56 ± 0.06	12.57 ± 0.10	16.65 ± 0.11	21.58 ± 0.12	27.26 ± 0.21

**Table 6:** Results of antiviral/cytotoxicity testing [66].

Entry	Compound	% Reduction in Number in Plaques	Minimum Antiviral Concentration (mg/mL)	Cytotoxicity (CD_50_) (mg/mL)
1	(+)-aphidicolin (**28**)	100	0.005	0.20
2	(+)-stemodin (**1**)	67	0.02	0.04
3	(−)-7α-hydroxystemodin (**32**)	33	0.1	0.20
4	(−)-7β-hydroxystemodin (**33**)	none	-	0.07
5	14α-hydroxystemodin (**34**)	27	0.1	0.15
6	(−)-19-hydroxystemodin (**35**)	none	-	>0.399
7	(−)-17,19-dihydroxystemodin (**36**)	none	-	>0.399
8	(−)-18-hydroxystemodin (**37**)	67	0.1	0.20
9	16,18-dihydroxystemodin (**38**)	none	-	0.07
10	(−)-8β-hydroxystemodin (**39**)	93	0.1	0.30
11	(−)-8β,18-dihydroxystemodin (**40**)	none	-	0.66
12	7β,8β -dihydroxystemodin (**41**)	none	-	0.04
